# HuR modulation counteracts lipopolysaccharide response in murine macrophages

**DOI:** 10.1242/dmm.050120

**Published:** 2023-03-29

**Authors:** Isabelle Bonomo, Giulia Assoni, Valeria La Pietra, Giulia Canarutto, Elisa Facen, Greta Donati, Chiara Zucal, Silvia Genovese, Mariachiara Micaelli, Anna Pérez-Ràfols, Sergio Robbiati, Dimitris L. Kontoyannis, Marilenia De Matteo, Marco Fragai, Pierfausto Seneci, Luciana Marinelli, Daniela Arosio, Silvano Piazza, Alessandro Provenzani

**Affiliations:** ^1^ Department of Cellular, Computational and Integrative Biology (CIBIO), University of Trento, Via Sommarive 9, 38123 Trento, Italy; ^2^ Department of Chemistry, University of Milan – Statale, Via Golgi 19, 20133 Milan, Italy; ^3^ Department of Pharmacy, University of Napoli Federico II, Via D. Montesano 49, 80131 Napoli, Italy; ^4^ International Centre for Genetic Engineering and Biotechnology (ICGEB), Via Padriciano 99, 34149 Trieste, Italy; ^5^ Department of Chemistry ‘Ugo Schiff’, University of Florence, Sesto Fiorentino 50019, Italy; ^6^ Giotto Biotech, S.R.L, Sesto Fiorentino, Florence 50019, Italy; ^7^ Biomedical Sciences Research Centre ‘Alexander Fleming’, Institute of Fundamental Biomedical Research, Vari 16672, Greece; ^8^ Division of Genetics, Development and Molecular Biology, School of Biology, Aristotle University of Thessaloniki, 54124 Thessaloniki, Greece; ^9^ IRBM Promidis S.r.l., Pomezia, Rome 00071, Italy; ^10^ Centre for Magnetic Resonance (CERM), University of Florence, via Sacconi 6, 50019 Sesto Fiorentino, Florence, Italy; ^11^ Istituto di Scienze e Tecnologie Chimiche (SCITEC) ‘Giulio Natta’, Consiglio Nazionale delle Ricerche (CNR), Via C. Golgi 19, I-20133 Milan, Italy

**Keywords:** ELAVL1, HuR, LPS, RIP-seq, Anti-inflammatory agents, Tanshinone mimics

## Abstract

Lipopolysaccharide (LPS) exposure to macrophages induces an inflammatory response, which is regulated at the transcriptional and post-transcriptional levels. HuR (ELAVL1) is an RNA-binding protein that regulates cytokines and chemokines transcripts containing AU/U-rich elements (AREs) and mediates the LPS-induced response. Here, we show that small-molecule tanshinone mimics (TMs) inhibiting HuR–RNA interaction counteract LPS stimulus in macrophages. TMs exist in solution in keto-enolic tautomerism, and molecular dynamic calculations showed the ortho-quinone form inhibiting binding of HuR to mRNA targets. TM activity was lost *in vitro* by blocking the diphenolic reduced form as a diacetate, but resulted in prodrug-like activity *in vivo*. RNA and ribonucleoprotein immunoprecipitation sequencing revealed that LPS induces a strong coupling between differentially expressed genes and HuR-bound genes, and TMs reduced such interactions. TMs decreased the association of HuR with genes involved in chemotaxis and immune response, including *Cxcl10*, *Il1b* and *Cd40*, reducing their expression and protein secretion in primary murine bone marrow-derived macrophages and in an LPS-induced peritonitis model. Overall, TMs show anti-inflammatory properties *in vivo* and suggest HuR as a potential therapeutic target for inflammation-related diseases.

## Teaser

**Summary:** This extensive study provides progress in the development of synthetic modulators of HuR functions, their genome-wide mechanism of action and their activity as anti-inflammatory agents.

## INTRODUCTION

RNA-binding proteins (RBPs) play a pivotal role in the regulation of gene expression in eukaryotes by exploiting RNA–protein and protein–protein interactions (Glisovic et al., 2008; Turner and Díaz-Muñoz, 2018). RBPs' aberrant expression, their modulation or their mislocalization lead to the insurgence of complex phenotypes and diseases (Lukong et al., 2008; Hong, 2017; Gebauer et al., 2021). Therefore, targeting and modulating the activity of RBPs associated with various pathologies represents a new promising therapeutic strategy (D'Agostino et al., 2019). In this context, human antigen R (HuR; official name ELAVL1) is among the most widely studied RBPs. It belongs to the ELAVL protein family, is ubiquitously expressed in human tissues and is highly conserved during mammalian evolution (Ma et al., 1996; Zucal et al., 2015; Assoni et al., 2022). HuR binds AU/U-rich elements (AREs), located mainly in the 3′-UTRs of coding and non-coding RNA. ARE sequences are found in 7% of the human mRNAs, coding for proteins involved in key cellular processes such as immune response and inflammation (Atasoy et al., 1998; Katsanou et al., 2005; Yiakouvaki et al., 2012; Kafasla et al., 2014), cell division and proliferation (Wang et al., 2001; Giammanco et al., 2014), angiogenesis (Levy et al., 1998; Chang et al., 2014; Tang et al., 2002), senescence (Wang et al., 2001; Marasa et al., 2010; Pang et al., 2013) and apoptosis (Giammanco et al., 2014; Abdelmohsen et al., 2007; Izquierdo, 2008). A strong regulatory role for HuR is demonstrated by the fact that ∼90% of mRNAs coding for cytokines and chemokines contain repeated ARE sites in their 3′-UTR (Fan and Steitz, 1998; Fan et al., 2005; Seko et al., 2006). Consequently, HuR aberrant expression or subcellular distribution is connected to diseases such as cancer and immune pathologies (Schultz et al., 2020; Abdelmohsen et al., 2010). Thus, HuR should represent a valuable therapeutic target. HuR structure is characterized by three RNA recognition motifs (RRMs). RRM1 and RRM2 strictly cooperate, with RRM1 primarily responsible for RNA binding, and RRM2 and the interdomain linker significantly increase the RNA-binding affinity of both RRMs. In the presence of RNA, RRM1 and RRM2 undergo conformational changes, assume a closed shape and form a positively charged cleft responsible for RNA binding (Wang et al., 2013). RRM3 is known to bind mRNA polyA tails and promote protein oligomerization, but also contributes to RNA binding (Pabis et al., 2019).

Among HuR inhibitors reported so far (Assoni et al., 2022), tanshinone mimics (TMs) are synthetic compounds interfering with HuR activity, and their structure–activity relationships (SARs) have been described (Manzoni et al., 2018). They modulate HuR activity by competing with HuR–RNA complex formation, interacting with the protein in the region responsible for RNA binding (Manzoni et al., 2018; Lal et al., 2017). Consequently, TMs inhibit HuR regulation of cancerogenic mRNAs, showing anti-tumoral traits. Here, we explored the inhibitory activity of TMs on HuR-mediated response to inflammatory stimuli, specifically driven by lipopolysaccharide (LPS). HuR upregulates inflammation processes through several mechanisms, such as impeding Toll-like receptor 4 (*TLR4*) mRNA degradation and stabilizing various inducible pro-inflammatory transcripts (e.g. *IFNG*, *TNF*, *IL6*) (Anderson, 2010). Photoactivatable ribonucleoside-enhanced crosslinking and immunoprecipitation (PAR-CLIP) experiments in primary macrophages challenged with an LPS inflammatory stimulus showed the existence of a complex post-transcriptional response driven by the engagement of the RBP tristetraprolin (TTP) and HuR (Sedlyarov et al., 2016). Here, we synthesized three TM derivatives and a diacetate prodrug to improve solubility and provide significant *in vivo* activity. The mode of action of such TMs in counteracting the LPS response during co-administration was elucidated at the genome-wide level, and led to the identification of inflammatory target mRNAs, such as *Cxcl10* and *Il1b*, the HuR binding of which is regulated by TMs. Moreover, the anti-inflammatory properties of TMs were shown *in vivo*, in an LPS-induced peritonitis mouse model.

## RESULTS

### TMs show a redox keto-enolic tautomerism

**Fig. 1.**
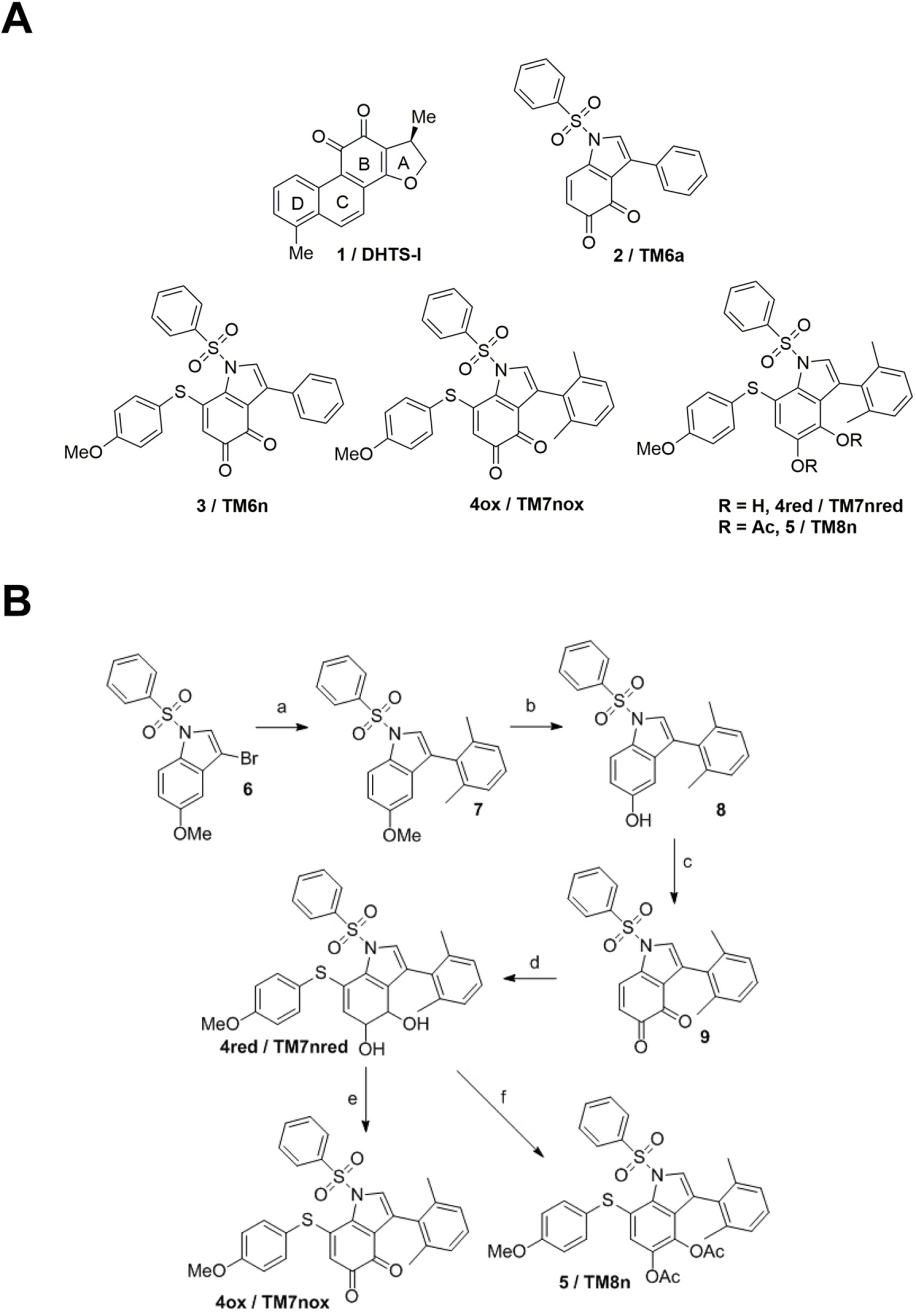
**Chemical structure and synthesis of tanshinone mimics (TMs).** (A) Chemical structure of standard DHTS-I 1 and unsubstituted TM6a 2 (top row), and of early lead 3/TM6n, dimethyl quinone 4ox/TM7nox, dimethyl diphenol 4red/TM7nred and dimethyl diacetate 5/TM8n (bottom row). (B) Synthesis of dimethyl quinone 4ox/TM7nox, dimethyl diphenol 4red/TM7nred and dimethyl diacetate 5/TM8n. Steps were as follows: (a) 2,6-dimethylphenyl boronic acid, aqueous Na_2_CO_3_, Pd(PPh_3_)_4_, 1,4-dioxane, 90°C, 16 h, Argon, 55% yield; (b) 1 M BBr_3_, dichloromethane (DCM), –78°C to 0°C, 3.5 h, Argon, 90% yield; (c) 1-Hydroxy-1,2-benziodoxol-3(1H)-one 1-oxide (IBX), dimethyl formamide (DMF), room temperature (r.t.), 3 h, 88% yield; (d) 4-methoxythiophenol, DMF, r.t., 1 h, Argon, 70% yield; (e) IBX, DMF, r.t., 30 min., 80% yield; (f) Ac_2_O, pyridine, DCM, r.t., 24 h, N_2_, 97% yield.

In earlier efforts, we identified naturally occurring dihydrotanshinone (DHTS)-I (Lal et al., 2017; D'Agostino et al., 2015) and a DHTS-inspired family of synthetic TMs, such as unsubstituted TM 2/TM6a (Fig. 1A) (Manzoni et al., 2018). We selected our most potent earlier TM 3/TM6n, (Fig. 1A), and we aimed to introduce two ortho substituents on the 3-phenyl ring of the 3-aryl-1,3-aza tanshinone system (4ox/TM7nox; Fig. 1A), to possibly increase solubility, as planarity disruption is known to lead to higher solubility of orto-substituted compounds (Luthe et al., 2007). Furthermore, we explored alternative routes to higher bioavailability by reducing the orto-quinone to a more hydrophilic ortho-diphenolic compound (4red, TM7nred), and we acetylated the latter to yield a putative esterase-sensitive prodrug 5/TM8n, which could escape any quinone-driven metabolic instability before reaching its molecular target.

Compounds TM6n (3), TM7nox (4ox; quinone), TM7nred (4red; diphenol) and 5/TM8n were synthesized and profiled in biological and physicochemical assays. Working on the published synthesis of TM6n (Manzoni et al., 2018), we improved it to yield bis-orto-substituted TM 4/TM7nox, TM7nred and 5/TM8n (Fig. 1B) on a multi-gram scale. A standard Suzuki coupling protocol using 2,6-dimethylphenyl boronic acid led to desired 3-aryl 5-methoxy intermediate 7 (Fig. 1B) in poor yields (∼6%), likely due to the additional steric hindrance around the reaction site. Conversely, by switching to dioxane as a solvent and raising the reaction temperature (Fig. 1B, step a), we obtained target 3-aryl 5-methoxy intermediate 7 in moderate yields. Standard demethylation and oxidation of phenoxy intermediate 8 (Fig. 1B, steps b and c, respectively) led to 3-(2,6-dimethyl)-phenyl TM 9 in good yields. The reduced, diphenolic 7-functionalized target 4red/TM7nred was then obtained in good yields following a standard 1,4-Michael addition protocol on intermediate 9 [step d (Manzoni et al., 2018)]. A portion of diphenolic 4red was then oxidized to ortho-quinone 4ox/TM7nox using a standard IBX protocol (Fig. 1B, step e), while another portion was easily converted into the acetylated pro-drug derivative 5/TM8n by almost quantitative acetylation (Fig. 1B, step f).

We then investigated the TM7nox/TM7nred interconversion equilibrium – in particular, the ability of diphenolic TM7nred to spontaneously convert to quinonic TM7nox in various media. Proton nuclear magnetic resonance (^1^H-NMR) studies were performed, in which we observed that diphenolic TM7nred reached an equilibrium at room temperature (RT) with quinone TM7nox when dissolved in organic solvent (acetone-d_6_), reaching ∼35% conversion into the oxidized form in 48 h (Fig. S1A). The conversion rate of TM7nred into TM7nox increased when D_2_O (10% v/v) was added to the solvent, reaching ∼46% after 24 h (Fig. S1B). Conversely, the oxidized form TM7nox dissolved in acetone-d_6_ showed higher stability and limited (∼12%) conversion into diphenol TM7nred after 45 days/1.5 months. Thus, we can safely assume that the biologically active/HuR-binding orto-quinone TM form should be the major species in biological environments, in agreement with modeling data.

We then considered an alternative pro-drug approach (Rautio et al., 2018) to prevent metabolic transformations or off-target interactions due to such redox equilibration. In particular, by masking the orto-quinone of Michael adduct TM7nox as a diacetate (5/TM8n; Fig. 1A), we aimed at obtaining a putative prodrug incapable of binding to HuR that can be converted *in situ* to reduced TM7nred by esterases, after reaching its cellular or *in vivo* targets. This reduced TM – as determined by the preliminary redox equilibration studies reported above – should eventually be converted into biologically active quinone TM7nox after O_2_-promoted equilibration in cellular media.

Early lead TM6n, the redox couple TM7nox and TM7nred, and diacetate TM8n were tested to determine their solubility in suitable aqueous media for *in vitro* and *in vivo* testing. We initially planned kinetic solubility measurements [liquid chromatography–mass spectrometry (LC-MS)] in PBS with increasing quantities of biocompatible solubilizer excipient Kolliphor EL; unfortunately, the Kolliphor EL turned out to be unsuitable, as its UV spectrum covers the peaks of our TMs and prevents their quantitation. Thus, we replaced Kolliphor EL with dimethyl sulfoxide (DMSO), and we surprisingly characterized early lead TM6n as the most soluble TM (from 0.8 μM in PBS-5% DMSO to 4.2 μM with 20% DMSO), followed by TM7nred (from 1.5 μM in PBS-10% DMSO to 2.9 μM with 20% DMSO), TM7nox (from 0.41 μM in PBS-10% DMSO to 1.8 μM with 20% DMSO) and TM8n (0.2 μM in PBS-20% DMSO). A graphic summary of kinetic solubility up to 20% DMSO for TM6n, TM7nred, TM7nox and TM8n is provided (Fig. S2). Although such results were discouraging, we attempted to solubilize the TMs in the medium for *in vivo* testing (PBS, 5% DMSO, 20% Kolliphor EL) and observed a significantly different behavior. Namely, dimethylated TM7nred, TM7nox and TM8n could be completely dissolved, whereas TM6n could not as it precipitated in the buffer for *in vivo* testing. Therefore, we decided to further characterize our TMs, while prioritizing TM7n-related (TM7nred, TM7nox and TM8n) derivatives for *in vivo* administration.

### The ortho-quinone TM7nox is the active form of TM7 series and keeps HuR in a closed conformation

**Fig. 2.**
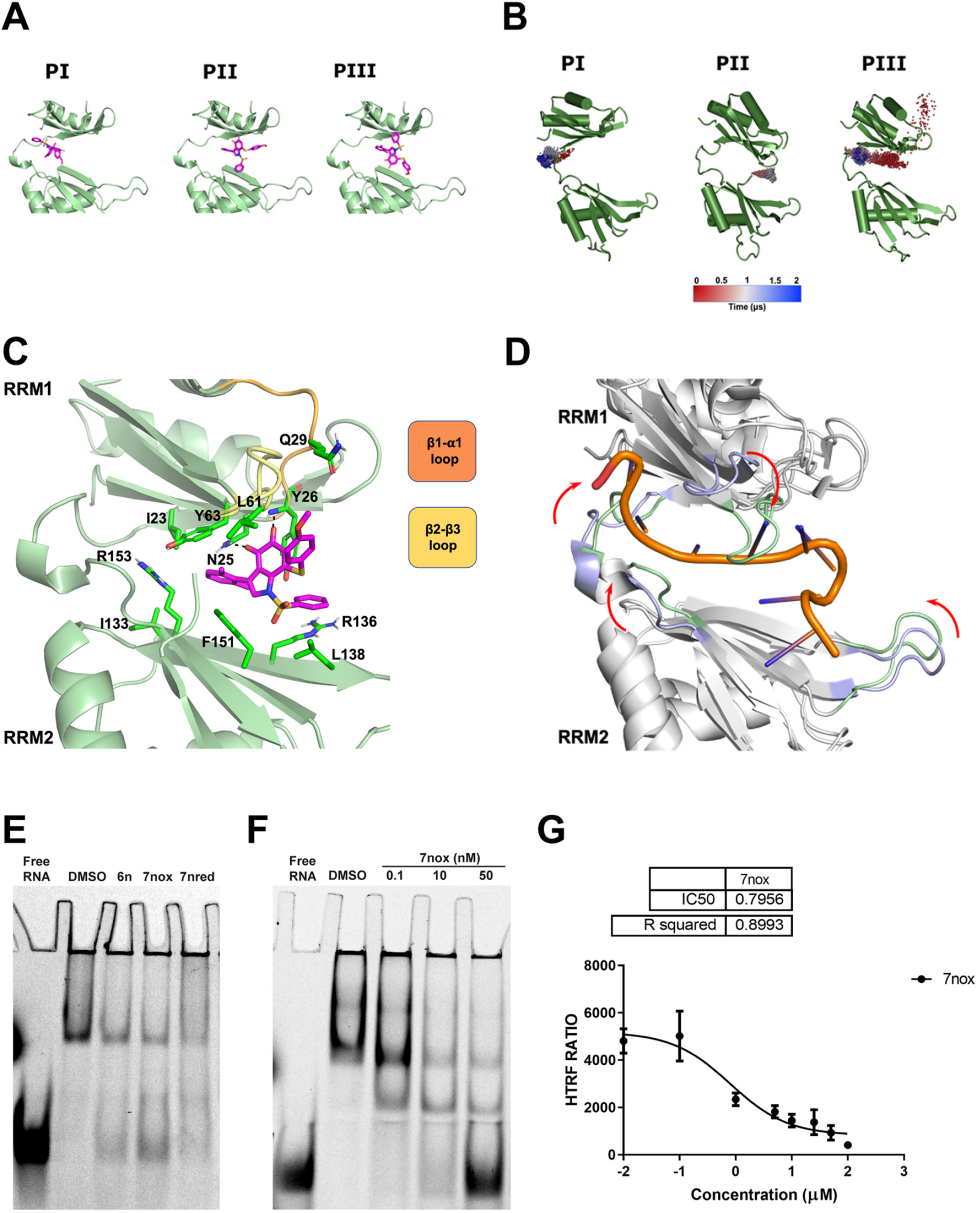
**TM7nox binds to HuR and disrupts HuR–RNA binding ability *in vitro*.** (A) Ligand-binding poses found by AutoDock4.2 and submitted to molecular dynamics (MD) simulations. The ligand is schematically shown in pink sticks, and HuR is represented in green. (B) Representation of the TM7nox exploration of the HuR cavity for each simulated pose. HuR is represented in green, and the ligand center of mass evolution during the trajectory is shown as colored spheres. (C) Theoretical TM7nox-binding mode PII, as suggested by our MD simulation. The ligand is shown in pink sticks and the protein in green. Main residues involved in interactions with the ligand are shown as green sticks. Nonpolar hydrogens are hidden for clarity. (D) Superposition of the HuR−RNA complex crystal structure with the final state of the dynamized PII. The secondary structures are depicted in gray; the loops are shown in light blue for the initial state and light green for the last frame of the MD simulation. (E) Representative RNA electromobility shift assay (REMSA) showing HuR–RNA binding impairment induced by TMs. rM1M2_HuR (3.7 nM) was incubated for 30 min with 1 nM 5′-DY681-labeled RNA probe alone, or together with DMSO used as control, or TM6n, TM7nox or TM7nred at 1 µM doses. (F) Representative REMSA showing TM7nox dose–response inhibition of the binding between 100 nM rM1M2_HuR and 1 nM 5′-DY681-labeled RNA probe. (G) Concentration–response analysis of TM7nox tested in HuR:RNA probe interaction assay. In a dose-dependent manner, TM7nox (0.01 μM, 0.1 μM, 1 μM, 5 μM, 10 μM, 25 μM, 50 μM, 100 μM) interferes with the binding between His-tagged recombinant M1M2 HuR protein (20 nM) and 5′-Bi-TNF ARE probe (50 nM). The calculated half-maximal inhibitory concentration (IC_50_) is 0.7956 µM, and data have been normalized to control (DMSO). Data fit nonlinear regression fitting curves according to a one-site binding model in GraphPad Prism. Plotted are mean±s.d. of three independent experiments.

Our earlier results show that DHTS-I (1) and our TM molecules, such as TM6a, bind to the β-platforms of the RRM1 and RRM2 domains, stabilizing a HuR closed conformation that hampers the accommodation of the binding mRNA (Manzoni et al., 2018). However, TM7n derivatives (TM7nred, TM7nox, TM8n) are more sterically hindered. Moreover, the planarity disruption induced by two ortho-methyl substituents on the 3-phenyl ring could partially change its stacking surface with respect to the previous synthesized TMs, so that a different accommodation within HuR must be considered. Thus, we investigated the molecular binding mode of TM7nox, the active and prevalent molecular species in solution. Molecular docking experiments were carried out using AutoDock4.2 (see Materials and Methods), allowing TM7nox to explore the entire interdomain space. Given the large region explored and the smaller size of TM7nox compared to mRNA, AutoDock did not find a unique binding mode. Thus, we focused on the lowest energy pose and on the poses representing the most populated clusters, referred to from now on as PI, PII and PIII. All three poses occupy the RRM1-RRM2 interdomain region and differ by a diverse closeness to the hinge region (Fig. 2A). Particularly, as for PI, TM7nox is placed closer to the hinge loop, resembling the binding mode found for TM6a (Manzoni et al., 2018). As for PII and PIII, TM7nox occupies the RRM1-RRM2 interdomain region in a more central area (less close to the hinge loop). To assess the relative stability of each pose, 2 µs-long molecular dynamics (MD) simulations were performed. The time evolution of TM7nox during the trajectories is graphically represented in Fig. 2B, where the TM7nox center of mass is shown as a sphere colored according to the simulation time [from red (initial poses) to blue (final poses)]. Plots of the root-mean-square deviation (RMSD) as a function of time with respect to the first step are shown in Fig. S3. The MD simulation of PI shows that TM7nox remains in the hinge region, although it changes its orientation over time because of the flexibility of the loop, which makes it difficult to retain stable interactions with neighboring residues. Indeed, the position of the center of mass during the simulation suggests that TM7nox explores different possible arrangements in the hinge loop (Fig. 2B). Despite this, the ligand stays in this binding region for the entire trajectory, as clearly shown by the RMSD plot (see Fig. S3). Similarly, in the PIII case, TM7nox once more accommodates in the hinge loop region, trying to establish stable contacts with the loop residues, after an important ligand reorientation (after ∼500 ns). Conversely, the PII pose seems to achieve clear and stable receptor interactions (Fig. 2B,C). In fact, the ortho-quinone oxygens are involved in two tight hydrogen bonds with the N25 side chain amide and with the Y26 NH backbone (see Fig. S4). Moreover, a ‘cage’ of aromatic residues (Y26, Y63 and F151) locks the central indole ring position during the simulation (Fig. 2C; Fig. S5). As for the dimethyl-phenyl moiety, it is buried in the interdomain cavity and is oriented toward the hinge loop, establishing an amide-π interaction with N25 and other hydrophobic contacts with Y63, I23, I133, F151 and R153 residues. The phenylsulfonyl and 4-methoxyphenylthio substituents rearrange themselves so that the former is kept in place for the whole simulation time by a strong cation-π interaction with R136 and other hydrophobic contacts within the RRM2 β-barrel (F151 and L138; the latter plunges between the β2-β3 and the β1-α1 loops of the RRM1 domain, contacting the L61 and Q29 residues). Noteworthily, looking at the superposition of the HuR−RNA complex crystal structure on the final state of the dynamized PII pose (Fig. 2D), the secondary structures are basically conserved during the simulation, while the loops surrounding the RNA binding cavity fold around the ligand, in order to dramatically reduce the available buried surface area between the two RRM domains. In particular, the β2-β3 loop flaps in the region where the A7-RNA base is located (Fig. 2D). Thus, in the PII final pose, TM7nox stabilizes HuR in a conformational state that hampers the accommodation of a binding RNA sequence. Our results suggest that, for TM7nox, although binding modes close to the HuR hinge loop are plausible and were found by both docking and 2 µs MD simulations, the center of the cavity made up by the beta-sheets of the two RRM domains is the only receptor region to establish stable contacts with the ligand moieties. In fact, the most significant difference between the PI-PIII binding modes and PII is, indeed, the lack in the former poses of two permanent H-bond interactions established by the quinone group. Finally, in line with experimental data (see Fig. S6A), molecular docking demonstrated that the binding of TM8n (prodrug-inactive derivative of TM7nox) to HuR is strongly unfavored. In fact, all its binding poses are higher in energy in comparison with TM7nox, and there is a failure in finding a convergence among the obtained poses (most clusters are populated by a single pose), suggesting that it is hard to find a reliable accommodation for TM8n. In summary, owing to chemical differences between TM7nox and TM6a, they bind HuR in a slightly different mode. Nonetheless, molecular modeling strongly suggests that they share the same mechanism of action, stabilizing an HuR closed conformation unable to accommodate the mRNA.

### Ortho-dimethyl TMs interfere with the HuR–RNA complex

The first two HuR domains were recombinantly (rHuR) produced in *Escherichia coli* BL21 and incubated with a 26 nucleotide (nt)-long, ARE-containing RNA probe linked by 5′ to the DY681 fluorophore to form the protein–RNA complex. RNA electromobility shift assays (REMSAs) revealed that TM6n, TM7nox, TM7nred and TM8n interfere with the binding of the protein with the RNA probe, and non-denaturing gel electrophoresis did not suggest the inhibition of protein dimerization (Fig. 2E,F; Fig. S6A,B). Such results are coherent with computational estimations and analytical studies, owing to the expected compatibility of ortho-dimethyl substitutions on the TM6n scaffold with HuR binding, and with the redox quinone-diphenol equilibrium, providing reduced TM7nred with the ability to disrupt the HuR–RNA complex via its conversion to TM7nox in biological media and the expected negative effect of the diacetate functionalization on HuR binding. To measure the disrupting ability of HuR–mRNA complex by TM7nox, we set up a homogenous time-resolved fluorescence (HTRF) assay. We used biotinylated 26-nt single-stranded RNA (ssRNA) probes containing the AU-rich elements of the *TNFα* (*TNF*) gene (Bi-TNF) to determine the hook point of the assay (D'Agostino et al., 2013) (Fig. S6C). We then titrated the protein–RNA complex with increasing concentrations of TM7nox and obtained a half-maximal inhibitory concentration (IC_50_) of 0.79 µM (Fig. 2G). Then, the activities of TM6n and TM7 derivatives in counteracting HuR were investigated in macrophages during LPS stimulation, a model in which HuR is known to regulate the cell response (Chellappan et al., 2022; Ostareck and Ostareck-Lederer, 2019; Lin et al., 2006).

### TM7nox reduces inflammatory and chemotaxis response induced by LPS in murine macrophage RAW 264.7 cell line

**Fig. 3.**
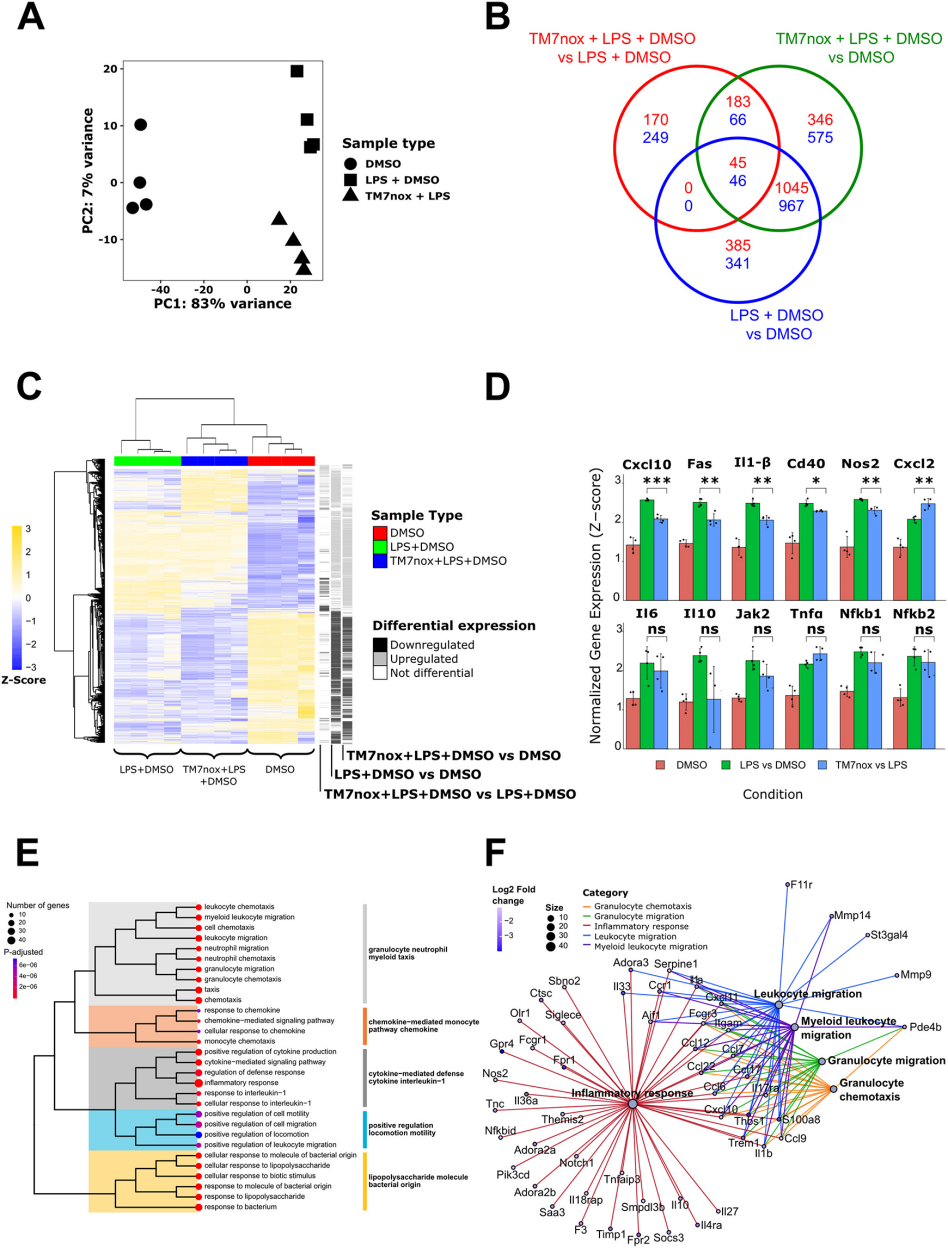
**RNA-seq analyses reveal that TM7nox modulates lipopolysaccharide (LPS)-induced response.** (A) Principal component analysis (PCA) of the 12 samples. PC1 shows 83% variance and PC2 7%. Each dot represents a DMSO sample, each triangle is a sample treated with DMSO+LPS, and each square is a DMSO+LPS+TM7nox-treated sample. Every condition groups together with the same type samples, and it can be observed that the effect of TM7nox separates DMSO+LPS from DMSO+LPS+TM7nox conditions. (B) Venn diagram of differentially expressed genes (DEGs); the numbers in each circle represent the number of DEGs between the different comparisons while the ones overlapping are for mutual DEGs (DMSO+LPS+TM7nox versus DMSO+LPS in red, DMSO+LPS+TM7nox versus DMSO in green, DMSO+LPS versus DMSO in blue). (C) Heatmap of *z*-score of differential genes across different samples, each grouping together with its own sample type (red, DMSO; green, DMSO+LPS; blue, DMSO+LPS+TM7nox). The track on the right shows differential gene expression between different comparisons (black, downregulated DEGs; gray, upregulated DEGs; white, no changes in DEGs). ‘Average’ was used as clustering method and ‘correlation’ for clustering distance of both rows and columns. (D) Bar plot of *z*-score of key genes across different samples (red, DMSO; green, DMSO+LPS; blue, DMSO+LPS+TM7nox). *P<0.05, **P<0.01, ***P<0.001; ns, not significant (Welch's *t*-test). The whiskers above/below the bars extend from the upper/lower quartile to the highest/lowest actual value that is within the [75th percentile±1.5×(interquartile range)]. (E) Tree plot of enriched terms deriving from the 249 downregulated genes. Each dot represents an enriched term, colored according to *P*-adjusted values, spanning from red to blue. Terms' dimensions are relative to the number of genes found to be enriched in that category. The subclusters and their names, visible on the right, are highlighted with a specific color. (F) Network visualization of the top-five-ranking Gene Ontology (GO) pathways for the 249 downregulated genes. Each pathway is represented by a gray dot and highlighted with a particular color; each gene is connected to the pathway it belongs to. Genes are represented in dots ranging from blue to white according to their log2FC values; the sizes of the pathway dots depends on the number of genes enriched for the pathway itself.

We evaluated the toxicity of TMs in the RAW 264.7 murine macrophage cell line and in bone marrow-derived macrophages (BMDMs) to better predict TM effects *in vivo*. BMDMs were harvested from 6- to 12-week-old C57BL6/j wild-type mice and, after 1-week *in vitro* differentiation of the monocytes into macrophages with L929 supernatant (Kontoyiannis et al., 1999), they were treated with TM6n, TM7nox, TM7nred and TM8n for 24 h. TMs seemed to show higher toxicity in primary cultures than in RAW 264.7 cells, considering that their IC_50_ is lower than 2 µM in BMDMs and higher than 10 µM in RAW 264.7 cells (Fig. S7A,B). To investigate the ability of TMs to modulate the macrophage response to inflammatory stimuli, we challenged RAW 264.7 cells with LPS (1 µg/ml) for 6 h. We chose TM7nox as a reference compound to evaluate its ability to counteract LPS-induced response, as the ortho-quinone form is the one interacting with HuR. We measured the abundance of transcripts after LPS treatment, and the ability of TM7nox to disrupt the interaction of HuR with its target mRNAs, by employing a transcriptome-wide approach of RNA preparations from untreated cells, LPS-treated cells and LPS+TM7nox-treated cells. Principal component analysis (PCA) showed that principal component 1 (PC1) and principal component 2 (PC2) explained most of the data variability, with 83% of variance associated with PC1 and 7% of variance associated with PC2. The effect of LPS treatment on transcriptome changes separating LPS-untreated from LPS-treated samples can be appreciated along the PC1 axis. LPS-treated groups segregated along PC2, which instead identified the effect of TM7nox on LPS-treated cells (Fig. 3A). Therefore, LPS is the major modulator of gene expression changes, but, within this context, TM7nox modulates the LPS cell response to a significant extent.

Indeed, LPS triggered a strong response by inducing 2829 differentially expressed genes (DEGs) with respect to the vehicle (DMSO; 1354 with logFC<−1, 1475 with logFC>1, Table S1). Co-treatment of LPS with TM7nox still induced a strong response, as evaluated by the number of DEGs (3273, 1654 downregulated and 1619 upregulated; Table S1). The number of genes commonly regulated by LPS, and by LPS–TM7nox co-treatment, accounted for (1045+45) upregulated+(967+46) downregulated genes (Fig. 3B; Fig. S8A). This ensemble of genes is modulated coherently in both treatments, represents the strong majority of DEGs, and can be considered as an LPS-induced response that is not affected by TM7nox. The strong effect of LPS can be also observed in the heatmap of DEGs in Fig. 3C, in which unsupervised clustering clearly separated LPS-treated samples from control samples. However, TM7nox samples organized as a subcluster within the larger LPS branch (Fig. 3C). Functional annotation identified several Gene Ontology (GO) terms related to the inflammatory process as the response to LPS, interferon γ and β, NF-κB activation and chemotaxis among upregulated genes; among the downregulated genes, we found several GO terms related to DNA replication and cell cycle (Table S2). Among the top upregulated DEGs, we found *Cxcl10*, *Fas*, *Il1b*, *Cd40*, *Nos2*, *Cxcl2*, *Il6* and *Il10* (Fig. 3D). These pathways and genes are widely recognized as canonically activated by LPS in RAW 264.7 cells, validating our experiment (Ostareck and Ostareck-Lederer, 2019; Tiedje et al., 2012). Three subgroups of regulated genes could also be identified (Fig. 3B), i.e. genes regulated by LPS/TM7nox versus DMSO, by LPS versus DMSO and by LPS/TM7nox versus LPS (Table S1). We focused our attention on the last subgroup, i.e. emerging categories from upregulated DEGs modulated by TM7nox in the presence of LPS (170 DEGs). Functional annotation of this group highlighted enrichment of cation transmembrane transporter activity genes (Table S3). Among the downregulated genes (249 DEGs), we observed a strong enrichment of categories related to the inflammatory response, cytokines (*Il1b*, *Cxcl10*, *Il10*, *Il19*, *Il33*), immune cell chemotaxis (*Ccl12*, *Ccl22*, *Ccl17*, *Ccl6*) and innate immune response (Table S3, Fig. S8B,C). The top-five-ranking GO pathways for the 249 downregulated genes of interest were further visualized with a tree plot (Fig. 3E) and network of the present DEGs (Fig. 3F). These results indicate that LPS-induced cellular response is not abolished by TM7nox, but rather modulated and mitigated. Specifically, TM7nox modulates the inflammatory response, downregulating the expression of important cytokines (*Il1b*, *Cxcl10*) and mainly influencing the expression of genes involved in cell chemotaxis. The modulation of LPS-induced response is further demonstrated by the presence of an exclusive LPS response that is absent during TM7nox co-treatment.

### TM7nox disrupts HuR interaction with selected ARE-containing transcripts

**Fig. 4.**
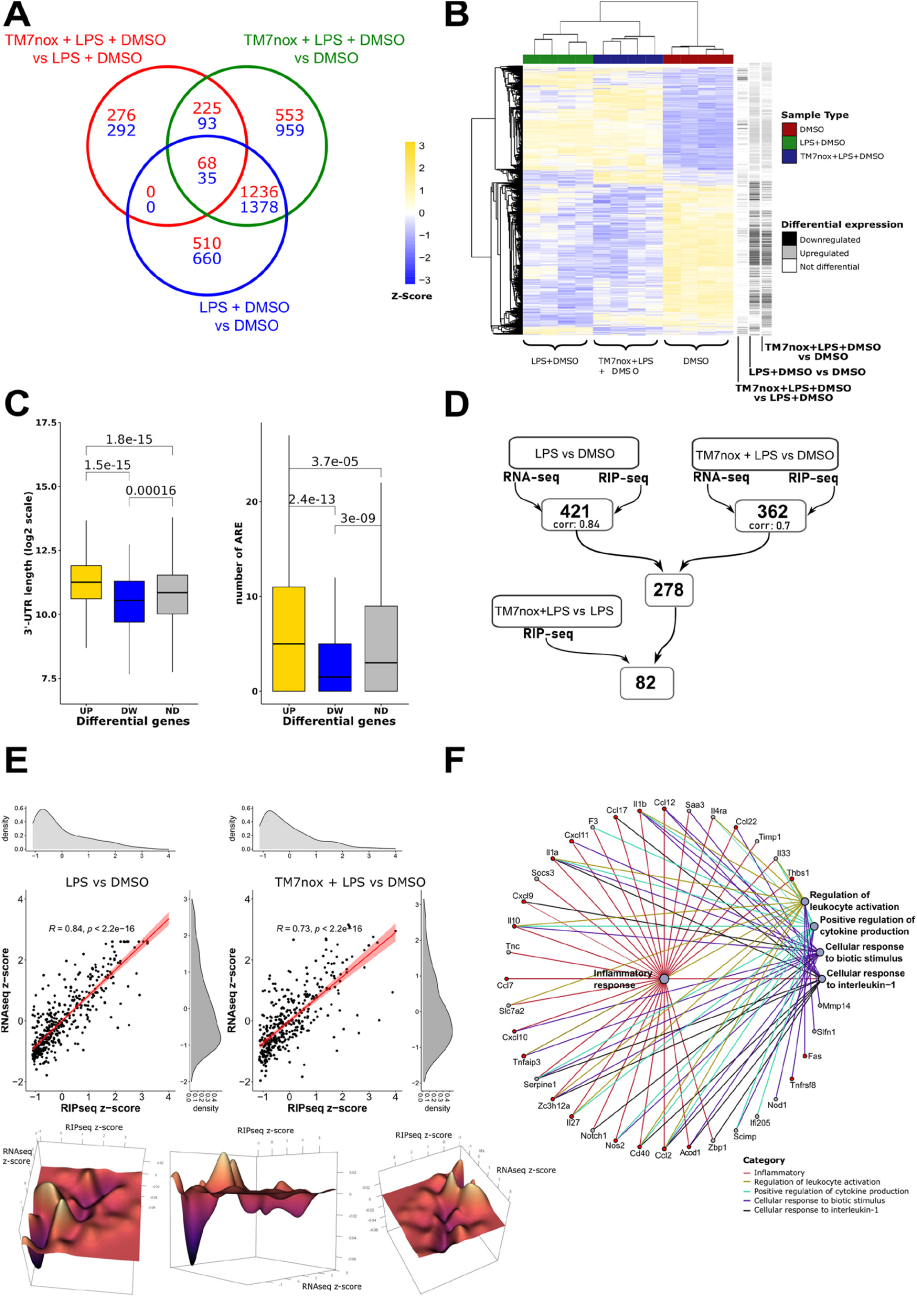
**Ribonucleoprotein immunoprecipitation followed by sequencing (RIP-seq) analysis reveals that TM7nox displaces the RNA targets from HuR.** (A) Venn diagram of DEGs; the numbers in each circle represent the number of DEGs between the different comparisons while the ones overlapping are for mutual DEGs (DMSO+LPS+TM7nox versus DMSO+LPS in green, DMSO+LPS+TM7nox versus DMSO in blue, DMSO+LPS versus DMSO in red). (B) Heatmap of *z*-score of differential genes across different samples, each grouping together with its own sample type (red, DMSO; green, DMSO+LPS; blue, DMSO+LPS+TM7nox). The track on the right shows differential gene expression between different comparisons (black, downregulated DEGs; gray, upregulated DEGs; white, no changes in DEGs). ‘Average’ was used as clustering method and ‘correlation’ for clustering distance of both rows and columns. (C) (Left) Boxplot of 3′-UTR length of the upregulated and downregulated DEGs in the TM7nox and LPS co-treatment versus LPS and DMSO RIP-seq jointly with non-differential genes (ND). Upregulated genes are in yellow, downregulated genes are in blue, non-differential genes are in gray. Wilcoxon test was performed between the three classes. (Right) Boxplot of the number of AU/U-rich elements (AREs) of the upregulated and downregulated DEGs in the TM7nox and LPS co-treatment versus LPS and DMSO RIP-seq jointly with non-differential genes. Upregulated genes are in yellow, downregulated genes are in blue, non-differential genes are in gray. Wilcoxon test was performed between the three classes. (D) Workflow of the gene-filtering process starting from the LPS versus DMSO and TM7nox+LPS versus DMSO comparisons in both RNA-seq (*P-*adjusted<0.05, log_2_FCe>1) and RIP-seq (*P-*adjusted<0.05, log_2_FC>3), identifying two different subsets of genes (421 genes with correlation value of 0.84 for LPS versus DMSO and 362 genes with correlation value of 0.73 for TM7nox+LPS versus DMSO comparison). 278 genes were thus identified to be commonly shared between these two subsets. Moreover, of the 82 genes resulting from the filtering with the TM7nox+LPS versus LPS+DMSO downregulated RIP-seq comparison (*P-*adjusted<0.05), 20 were found to be of particular interest (Table 1). (E) (Left) Correlation between the RNA-seq *z*-score values and RIP-seq *z*-score values for the LPS and DMSO comparison. Each black dot represents a gene present in both experiments with a log2FC>1 for RNA-seq and log2FC>3 for RIP-seq. Regression line is depicted in red. Density plots of the distributions for each subset of values are shown on the right for RNA-seq *z*-score values and on the top for the RIP-seq *z*-score values. (Right) Correlation between the RNA-seq *z*-score values and RIP-seq *z*-score values for the TM7nox and LPS co-treatment versus DMSO comparison. Each black dot represents a gene present in both experiments with a log2FC>1 for RNA-seq and log2FC>3 for RIP-seq. Regression line is depicted in red. Density plots of the distributions for each subset of values are shown on the right for RNA-seq *z*-score values and on the top for the RIP-seq *z*-score values. (Bottom) 3D-rendered visualization from different angles of the difference between the correlation densities for LPS and DMSO comparison and TM7nox and LPS co-treatment versus DMSO comparison. On the axes, RNA-seq *z*-score values, RIP-seq *z*-score values and distribution density differences are shown. Color varies from purple to yellow according to the difference in the distribution values. (F) Network visualization of the top-five-ranking GO pathways of a subset of 82 genes of interest. Each pathway is represented by a gray dot and highlighted with a particular color; each gene is connected to the pathway it belongs to. A subset of 20 genes of interest is highlighted in red.

We performed a HuR ribonucleoprotein immunoprecipitation followed by sequencing (RIP-seq) experiment in RAW 264.7 cells to appreciate the effect of TM7nox on the modulation of gene expression response induced by LPS treatment, and its dependence on its biochemical activity as a disruptor of HuR/RNA binding. The enrichment fold change (FC) was calculated by comparing HuR-bound transcripts in each condition. We found a strong positive association of transcripts bound by HuR after LPS treatment (3887) in comparison to DMSO-associated transcripts (Table S1) (Lal et al., 2017). PCA indicated that cellular response to LPS strongly modifies the HuR-bound transcriptome, segregating LPS-treated versus untreated samples and justifying the observed 86% variance. PC2 described the effect of TM7nox on HuR-bound transcripts during LPS treatment (8%). In absolute values, both PCs showed similar variance than at the transcriptome level (Fig. S9A). DEG distributions in the considered comparison were visualized with a Venn diagram (Fig. 4A; Fig. S9B). Similarly, for the RNA-sequencing (RNA-seq) data, a strong effect of LPS can be observed in the heatmap of differentially enriched genes, in which the unsupervised clustering separated LPS-treated samples from control samples, with the subcluster of TM7nox samples separately organized within the larger LPS branch (Fig. 4B). As DHTS displaces transcripts with shorter 3′-UTR and with lower AREs than average (Lal et al., 2017), we evaluated these two parameters in the comparisons between TM7nox and LPS co-treatment versus LPS and LPS versus DMSO. We observed that HuR-bound enriched mRNAs contained longer 3′-UTRs than the downregulated ones as well as than those not differentially regulated (Fig. 4C, left). Moreover, the number of AU/U-rich regions present in HuR-bound mRNAs was higher than that in mRNAs that lost association or did not change (Fig. 4C, right; Table S4). Therefore, TM7nox displaces transcripts from HuR in a similar manner to DHTS. To gain more insights into the mechanism of action of TMs, we investigated the correlation between induced gene transcription and HuR association (Fig. 4D,E). We observed that 421 HuR-bound transcripts (log2FC>3) were also upregulated by LPS at the transcriptional level (log2FC>1). We observed a strong correlation (coefficient, 0.84; Fig. 4E, left) between these two gene ensembles, corroborating the hypothesis of a HuR-mediated LPS response. GO analyses of these genes mirrored the LPS response at the transcriptome level, highlighting categories related to the innate immunity response, cytokine activity and chemotaxis. Many relevant genes mediating these responses were found to be associated with HuR, such as chemokines (*Cxcl2*, *Cxcl10*), interleukins (*Il1a*, *Il1b*, *Il6*, *Il10*) and those encoding key regulators of immunity, such as the Cd40 antigen, Janus kinase 2 and nitric oxide synthase 2.

**Table 1.**
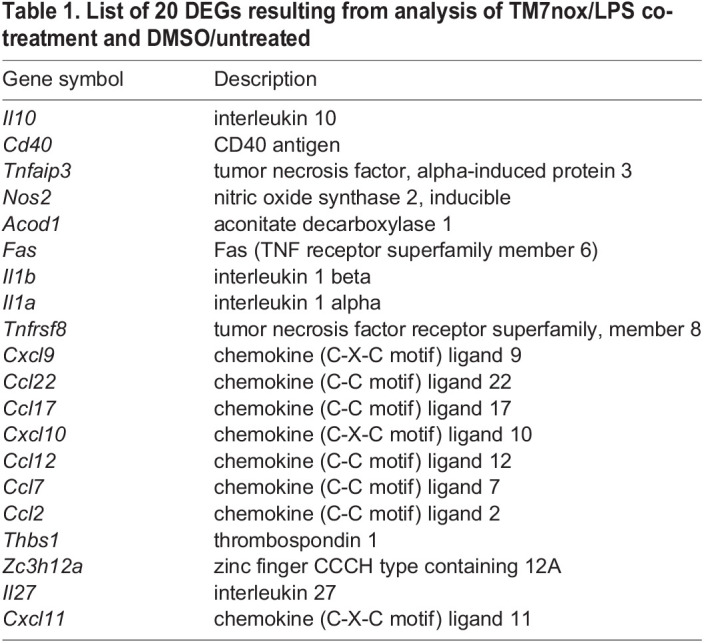
List of 20 DEGs resulting from analysis of TM7nox/LPS co-treatment and DMSO/untreated

By applying the same filtering process to TM7nox/LPS co-treated versus DMSO-treated samples, we found 362 upregulated genes, but we observed a decrease in the correlation coefficient to 0.73 (Fig. 4E, right), suggesting an uncoupling effect of TM7nox on the association of each mRNA with HuR and its expression level. The difference between the two correlation densities was calculated and rendered in 3D using Delaunay triangulation and Dirchlet tessellation (Fig. 4E, bottom). We found that 278 of the 362 genes were also present among the 421 transcripts of the previous LPS-treated dataset, but, despite an increase in the expression level and in HuR association compared to the DMSO/untreated level, 82 of these genes showed decreased association with HuR in the TM7nox/LPS co-treatment condition with respect to the LPS condition. Interestingly, 47 of 82 genes contained less AU-rich motif than the average, suggesting that the decreased association with HuR is dependent on the presence of the AU-rich motif (Table S1). GO analysis pointed to the inflammatory response as an over-represented category (Fig. 4F; Table S3). Such genes were coherently modulated with a similar trajectory by TM7nox/LPS co-treatment, with *Cxcl10*, *Il1b*, *Cd40*, *Fas*, *Nos2* (Fig. S9D) as notable examples. As the correlation coefficient between expression and mRNA–HuR association decreased from 0.84 to 0.73 in the presence of TM7nox, we can infer that the latter modulates the ability of HuR to bind specific mRNAs. Therefore, this set of genes can be considered a core set of LPS-induced genes for which association with HuR has been dampened by TM7nox.

### TMs affect mRNA expression of LPS-inducible genes and decrease binding of HuR to target RNAs

**Fig. 5.**
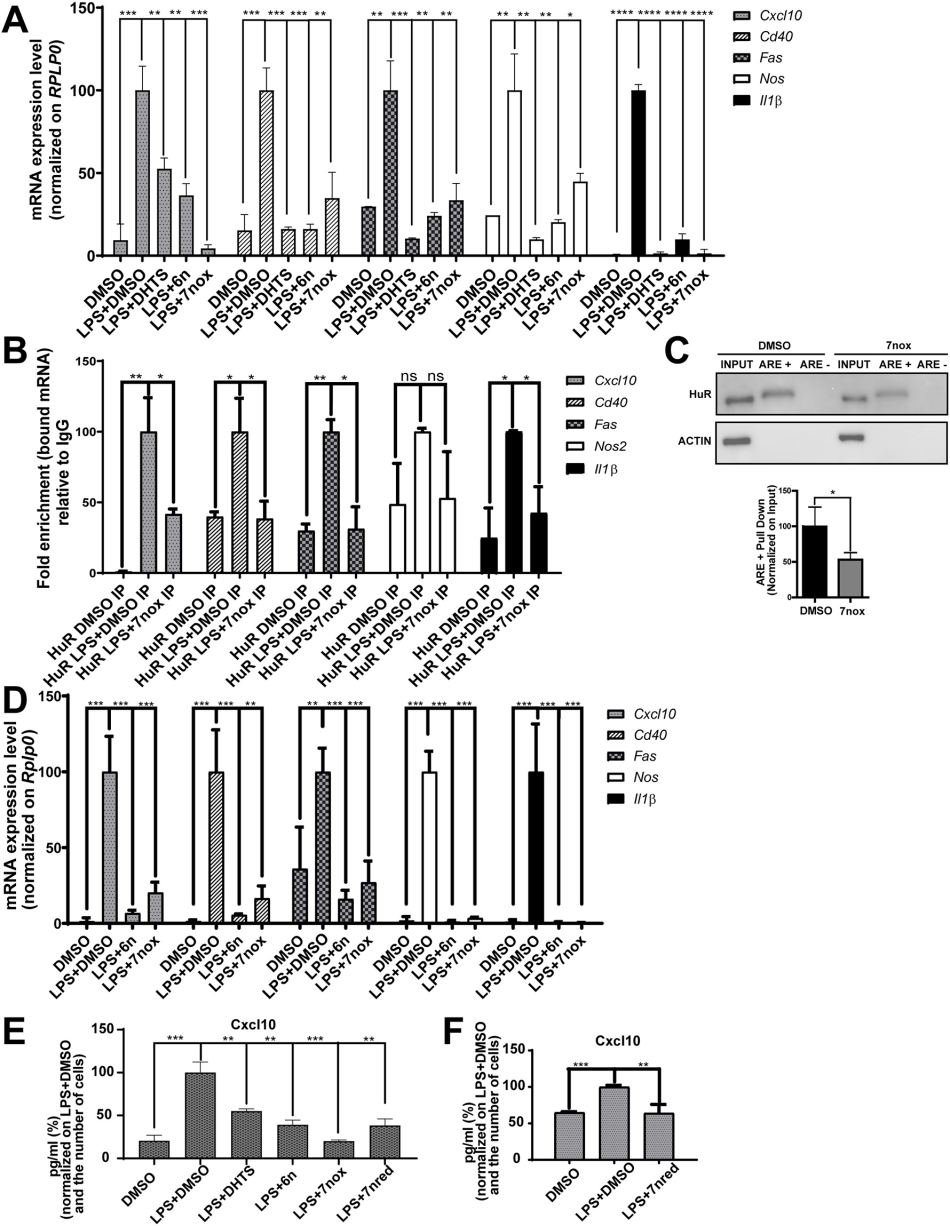
**TM7nox/TM7nred decrease the binding between HuR and identified targets, reducing their expression level and Cxcl10 secretion in RAW 264.7 cells and bone marrow-derived macrophages (BMDMs).** (A) RAW 264.7 cells were co-treated for 6 h with DHTS (5 µM), TM6n and TM7nox (10 µM), LPS (1 µg/ml) and DMSO as control. mRNA levels of *Cxcl10*, *Cd40*, *Fas*, *Nos2* and *Il1b* were assessed using qRT-PCR with *Rplp0* as housekeeping gene; data were normalized to LPS+DMSO condition. Data plotted as mean±s.d. of biological quadruplicate (*P<0.05, **P<0.01, ***P<0.001, ****P<0.0001; Welch's *t*-test). (B) TM7nox impairs the binding between HuR and identified targets. RIP-seq results were validated by RNA immunoprecipitation (RIP) followed by quantitative real-time PCR (qRT-PCR). RAW 264.7 cells were treated for 6 h with DMSO alone and LPS (1 µg/µl)+DMSO as controls, and LPS (1 µg/ml)+TM7nox (10 µM). Subsequently, cells were lysed, and RNA was precipitated with anti-HuR antibody [immunoprecipitation (IP)] and IgG isotype (IgG) as negative control. Changes in the mRNAs bound to HuR in the control or treatment were evaluated through qRT-PCR and normalized to the corresponding values obtained with IgG as negative control. The obtained numbers indicate the fold enrichment; experiments were performed in biological triplicate (*P<0.05, **P<0.01; ns, not significant; Welch's *t*-test). (C) Pull-down assays performed in RAW 264.7 cells pre-treated with TM7nox or DMSO as control for 6 h. Cell lysates were incubated for 1 h at 4°C with either biotinylated probe containing HuR consensus sequence, or probe not supposed to bound by HuR as a negative control. Precipitations of the probes were carried out with streptavidin beads, and HuR levels were detected by western blot analysis. HuR signal was quantified as the input (10%) and normalized to the DMSO sample. Data plotted as mean±s.d. of three independent experiments (*P<0.05; Welch's *t*-test). (D) BMDMs were co-treated for 6 h with TM6n and 7nox at 10 µM doses, LPS (1 µg/ml) and DMSO as control. mRNA levels of *Cxcl10*, *Cd40*, *Fas*, *Nos2 and Il1b* were assessed using qRT-PCR with *Rplp0* as housekeeping gene; data were normalized to the LPS+DMSO condition. Data plotted as mean±s.d. of biological quadruplicate (**P<0.01, ***P<0.001; Welch's *t*-test). (E) TM7nox, TM6n, DHTS and TM7nred treatment reduce Cxcl10 secretion in RAW 264.7 cell supernatants. Cxcl10 protein levels were measured with ELISA. RAW 264.7 cells were treated for 6 h with DMSO as control, LPS (1 µg/ml) plus DMSO or TM7nred (10 µM). Relative quantity of Cxcl10 pg/ml for each sample was measured according to the number of cells quantified through Crystal Violet assay. Data were normalized to LPS+DMSO as control, and numbers are expressed as a percentage. Data represent as mean±s.d. of biological triplicate (**P<0.01, ***P<0.001; Welch's *t*-test). (F) TM7nred treatment reduces Cxcl10 secretion in BMDM supernatants. Cxcl10 protein levels were measured with ELISA. RAW 264.7 cells were treated for 6 h with DMSO as control, LPS (1 µg/ml) plus DMSO or TM7nred (10 µM). Relative quantity of Cxcl10 pg/ml for each sample was measured according to the number of cells quantified through Crystal Violet assay. Data were normalized to LPS+DMSO as control, and numbers are expressed as a percentage. Data represent as mean±s.d. of biological quadruplicate (**P<0.01, ***P<0.001; Welch's *t*-test).

Thus, considering our sequencing results, to generalize the activity of TMs, we investigated the effect of three TMs as a co-treatment with LPS in murine RAW 264.7 macrophages at the single-gene level. We co-treated cells with the active quinone species TM6n, TM7nox (10 µM), DHTS (5 µM) and LPS (1 µg/ml), and evaluated the expression levels of *Cxcl10*, *Cd40*, *Fas*, *Nos2* and *Il1b* by quantitative real-time PCR (qRT-PCR) at 6 h post-treatment (Fig. 5A). Although LPS treatment induced the activation of the expression of several cytokines, TM6n, TM7nox and DHTS decreased their mRNA levels, which appeared to be significantly downregulated at 6 h post co-treatments. Notably, *Cxcl10*, *Cd40*, *Fas* and *Il1b* were significantly decreased, with *Nos2* showing a trend by treatment with TM7nox in HuR immunoprecipitation (IP) samples, suggesting that TMs are indeed able to reduce the HuR–mRNA interaction within the cell (Fig. 5B). To confirm the putative disruption of selected HuR–RNA complexes by TMs within cells, we performed RNA pull-down experiments using RAW 264.7 cells pre-treated for 6 h, focusing on TM7nox and using DMSO as a control. Cell lysates were incubated with a biotinylated probe containing a 3′ ARE sequence belonging to *TNFα* 3′-UTR, and a negative biotinylated control containing a sequence that is not recognized by HuR (ARE-Neg). After incubation for 2 h at 4°C, biotinylated probes were precipitated with streptavidin beads and HuR levels were assessed by western blot analysis (Fig. 5C). Indeed, the HuR band in the TM7nox-treated sample precipitated with the *TNFα* ARE sequence was significantly decreased (∼ 50%) compared to that in cells treated with DMSO and with lysates precipitated with a HuR-unreactive ARE probe. This suggests that TM7nox competes with target RNAs for HuR binding within cells. BMDMs were co-treated with LPS (1 µg/ml) and TM6n or TM7nox (10 µM) for 6 h. TM treatment almost blocked LPS-induced mRNA expression, showing higher efficacy in BMDMs compared to RAW 264.7 cells (Fig. 5D). TM7nox, TM6n and control DHTS also decreased the release of Cxcl10 protein from RAW 264.7 cells after 6 h of treatment and post-LPS induction, as measured by enzyme-linked immunosorbent assay (ELISA) (Fig. 5E). Interestingly, TM7nred showed equivalent efficacy of TM7nox at the protein level in RAW 264.7 cells (Fig. 5E) and decreased Cxcl10 secretion in BMDMs (Fig. 5F). Taken together, these results validate the RNA-seq and RIP-seq data and show the ability of TMs to interfere with LPS-induced, HuR-mediated gene expression.

### TMs partially mimic HuR silencing and do not modulate NF-κB translocation

**Fig. 6.**
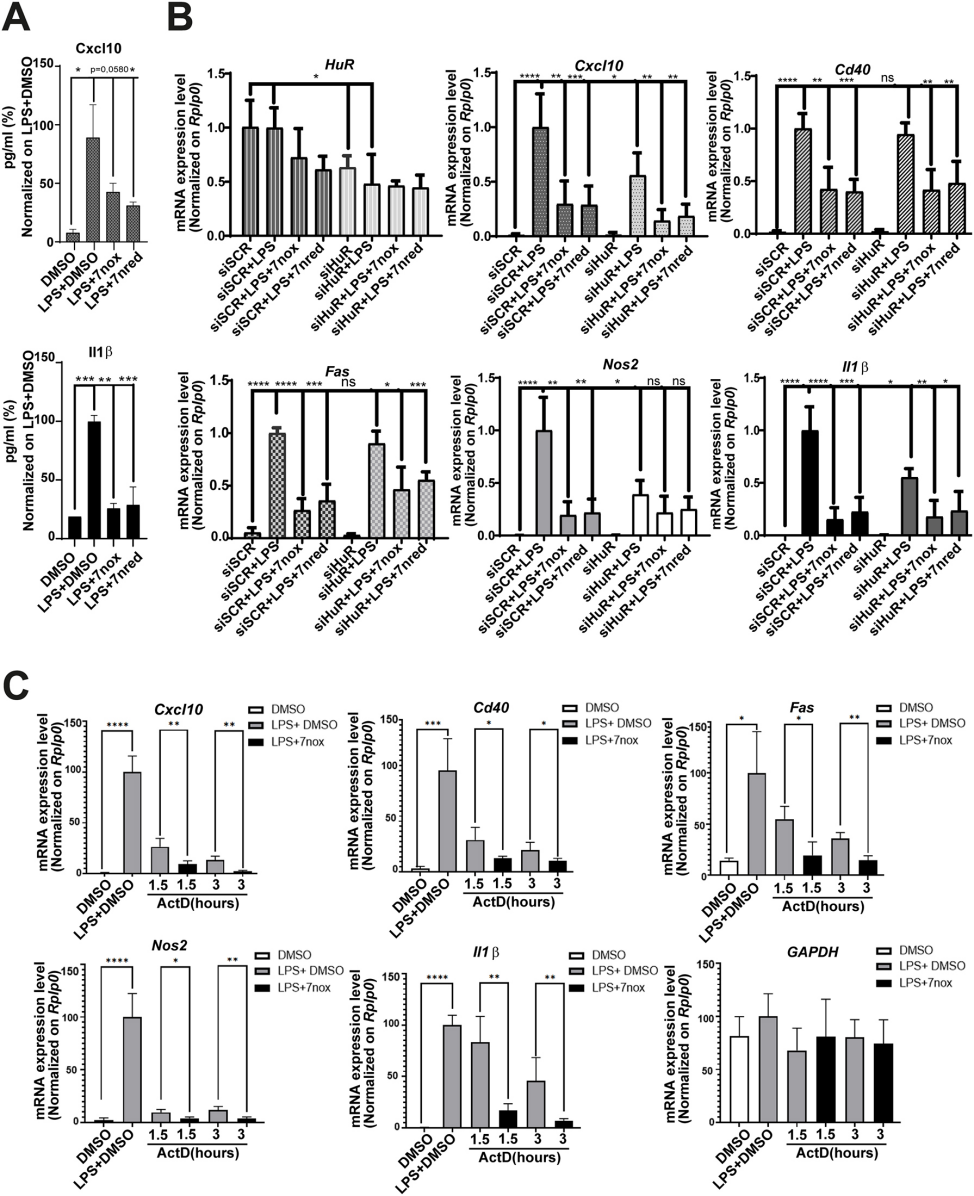
**TM7nox/TM7nred recapitulate partially HuR silencing without changing NF-κB translocation and HuR subcellular localization.** (A) TM7nox and TM7nred treatment reduce Cxcl10 (top) and Il1β (bottom) intracellular levels in RAW 264.7 cells. Protein levels were measured with ELISA. RAW 264.7 cells were treated for 6 h with DMSO as control, LPS (1 µg/ml) plus DMSO or TMs (10 µM). Respectively, 30 µg and 5 µg of cellular lysates were loaded to measure Cxcl10 and Il1β (pg/ml). Data were normalized to LPS+DMSO as control, and numbers are expressed as a percentage. Data represent mean±s.d. of biological triplicate (*P<0.05, **P<0.01, ***P<0.001; Welch's *t*-test). (B) qRT-PCR of target mRNAs in siSCR and siHuR, after 6 h of co-treatment with LPS 1 µg/ml plus DMSO, DMSO alone to control LPS stimulation, or 10 µM TM7nox or TM7nred in RAW 264.7 cells. Data plotted as mean±s.d. are from three independent experiments (*P≤0.05, **P≤0.01, ***P≤0.001, ****P<0.0001; ns, not significant; Welch's *t*-test). (C) TM7nox affects the transcription of LPS-induced cytokines. RAW 264.7 cells were co-treated with DMSO, LPS+DMSO, LPS+TM7nox for 3 h. Act-D (2.5 µM) was then added/administered for 1.5 h or 3 h. qRT-PCR was performed to quantify the remaining *Cxcl10*, *Il1b*, *Cd40*, *Fas*, *Nos2* and *Gapdh* mRNA levels. Data plotted as mean±s.d. of biological triplicate (*P≤0.05, **P≤0.01, ***P≤0.001, ****P<0.0001; Welch's *t*-test).

**Fig. 7.**
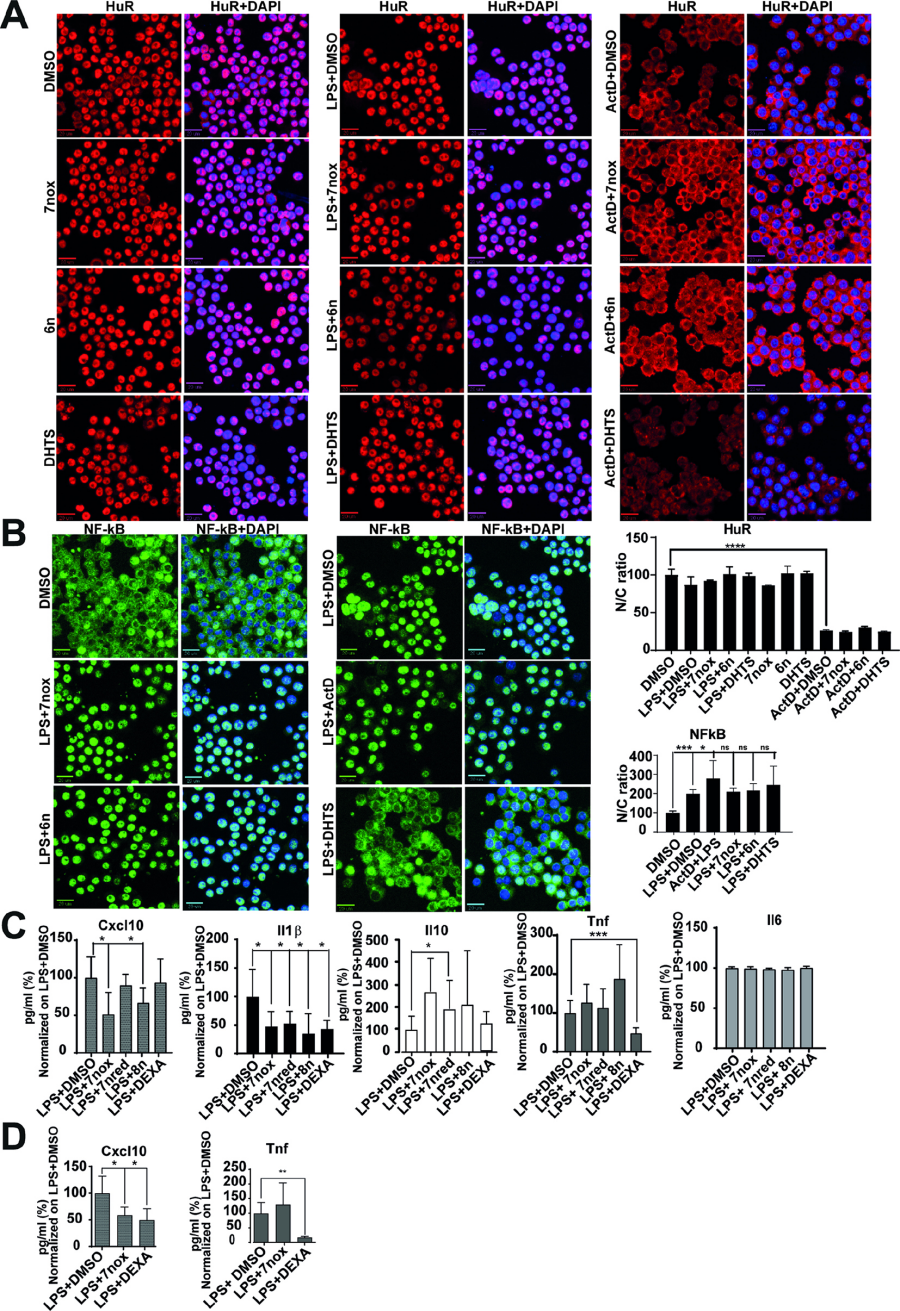
**TMs modulate Cxcl10 and Il1β secretion in LPS-induced peritonitis mouse models.** (A) Left and middle panels show representative immunofluorescence HuR localization after LPS administration, single-compound treatment and in combination with LPS (1 µg/ml). The right panel shows representative immunofluorescence showing that HuR cytoplasm accumulation induced by actinomycin D (ActD) does not change upon treatment with different TMs (10 µM). Cells were treated for 3 h in combination with ActD (2.5 µM). DMSO alone or in combination with ActD and LPS was used as control. In the graph, the ratio of HuR fluorescent signal between nucleus and cytoplasm (N/C) is plotted. For image acquisition (40× high-NA objective), Operetta was used, and evaluation was carried out by selecting 13 fields/well. The N/C ratio represents the mean±s.d. of single cells for every well (****P<0.0001; Welch's *t*-test). (B) Representative immunofluorescence showing that NF-κB nuclear translocation inside the cells induced by LPS does not change upon treatment with different TMs (10 µM). Cells were treated for 3 h in combination with LPS (1 µg/ml). To obtain a positive control given by high induction of NF-κB related to massive shuttling in the nucleus, we treated cells with 2.5 µM ActD; DMSO, either alone or with LPS, was used as negative control. In the graph, the ratio of NF-κB fluorescent N/C signal is plotted. For image acquisition (40× high-NA objective), Operetta was used, and evaluation was carried out by selecting 13 fields/well. The N/C ratio represents the mean±s.d. of single cells for every well (*P<0.05; ***P<0.0001; ns, not significant; Welch's *t*-test). (C) Levels of identified cytokines through Luminex analysis in sera from C57BL/6j wild-type mice after administration of LPS (150 μg/25 g of body weight) and TMs (40 mg/kg) or DMSO for 2 h. Data were normalized to LPS+DMSO and are expressed as mean±s.d. as a percentage of sex-balanced mouse group in which *n*=6-8 (*P<0.05, ***P<0.001; Welch's *t*-test). (D) Cxcl10 and Tnf levels measured with ELISA in the sera from C57BL/6j wild type mice after administration of LPS (150 μg/25 g of body weight) with TMs (40 mg/kg) or DMSO for 2 h. DMSO alone was used as a control for LPS inflammatory response insurgence, and LPS+DMSO was considered as the drug vehicle. Bar graphs show mean±s.d. from five mice per group (*P<0.05, **P≤0.01; Welch's *t*-test). Scale bars: 20 μm.

To investigate whether the activity of TMs in cells is due to inhibition of HuR, we compared the effect of TM7nred and TM7nox with that of HuR silencing by 48 h before LPS stimulation (6 h) in RAW 264.7 cells. In this case, we used both TM7n derivatives to show their biological equivalence in redox equilibration-compatible experimental protocols. Both compounds reduced the intracellular level of Cxcl10 and Il1b proteins, as measured by ELISA (Fig. 6A). LPS did not induce the expression of HuR (*Elavl1*) mRNA, while TMs reduced the expression of HuR mRNA both in DMSO/vehicle and under LPS stimulation. This effect may be ascribed to the autoregulatory mechanism of HuR on its own mRNA. HuR silencing (Fig. S10A) in DMSO or LPS condition reduced the expression of HuR mRNA to ∼50%, and both TMs did not show a significantly additive effect. The decrease in HuR did not reduce the expression level of *Cxcl10*, *Cd40*, *Fas*, *Nos2* and *Il1b* mRNAs in basal conditions. However, during LPS stimulation, *Cxcl10*, *Nos2* and *Il1b* expression levels were significantly decreased, whereas *Cd40* and *Fas* levels were not. Both TMs recapitulated the effect of HuR silencing during LPS stimulation for *Cxcl10*, *Nos2* and *Il1b*, but also decreased *Cd40* and *Fas* expression (Fig. 6B). The simultaneous treatment with either TM7n derivative and HuR silencing did not show additive effect to HuR silencing alone on the protein level of Cxcl10 and Il1b (Fig. S10B). We then evaluated the stability of *Cxcl10*, *Cd40*, *Fas*, *Nos2* and *Il1b* using two different protocols. In the first protocol, actinomycin D (ActD) was co-administered with TM7nox 3 h after LPS treatment; we evaluated the effect of TM7nox on the stability of the mRNAs irrespective of its transcriptional impact. Contrary to expectations, comparing the expression levels of target RNAs at 1.5 h after ActD, we observed a trend for increased stabilization (Fig. S10C). In a second experimental protocol, we administered TM7nox with LPS and added ActD after 3 h. By comparing the expression levels of target RNAs at 1.5 h and 3 h after ActD, we observed less mRNA in co-treated samples, suggesting a transcriptional impact of TM7nox during LPS co-treatment (Fig. 6C). Therefore, the effect of TM treatment resembles, but does not completely overlay with, HuR silencing, and the TM7n redox couple shows overlapping biological properties. Although a 3 h LPS treatment induces shuttling of HuR into the cytoplasm (Lin et al., 2006), co-treatment with our TMs did not counteract LPS-induced HuR shuttling. Similarly, TM6n and TM7nox (10 µM) did not induce HuR shuttling as previously observed in MCF-7 cells (Manzoni et al., 2018) and did not counteract ActD-induced massive shuttling of HuR (Fig. 7A). DHTS also did not modulate HuR localization. The LPS-induced NF-κB response was dampened by TM7nox, as suggested by functional analysis. To rule out that TMs directly inhibit the TLR4/NF-κB activity, we investigated whether NF-κB translocation into the nucleus was blocked by TMs, as this is the prerequisite for biological activity. Immunofluorescence experiments in RAW 264.7 cells (Fig. S10C,D) treated with TM6n or TM7nox for 3 h, alone or in combination with LPS (1 µg/ml), indicated that LPS stimulates NF-κB nuclear localization, while TMs did not show any significant counteracting or stimulating action on its translocation (Fig. 7B). DHTS partially reduced LPS-induced nuclear shuttling of NF-κB (Jang et al., 2006). Collectively, these data imply that TMs act independently from changing NF-κB cellular localization induced by LPS and are unable to stimulate HuR rescue in the nucleus as once inferred by drugs like ActD, suggesting once again that our TMs most likely function by modulating HuR–RNA binding activity.

### TMs effectively counteract LPS-induced inflammatory response *in vivo*

Finally, *in vivo* efficacy of TMs was evaluated in the LPS-induced peritonitis mouse model (Qin et al., 2016). Groups of six 8-week-old mice were co-treated with a sublethal dose of LPS and TM7red, TM7nox or its prodrug TM8n [intraperitoneal (i.p.), 40 mg/kg]; the redox couple was tested to evaluate the putative impact of different physicochemical properties on *in vivo* potency, while the putative prodrug TM8n represented a possible alternative route to *in vivo* efficacy. After 2 h, mouse blood was collected through cardiac puncture, serum was purified from plasma, and cytokines were detected either by ELISA or by Luminex technology. We detected an increase in Cxcl10, Il1b, Il6 and Tnfα cytokines in the mouse sera after LPS treatment, validating the efficacy of LPS in activating the inflammatory response. Cytokines were tested in batch by Luminex, and dexamethasone was used as a counteracting agent to LPS-induced inflammation. During co-treatment of LPS with TMs, Cxcl10 and Il1b levels decreased in sera derived from treated mice compared to those in sera from control mice (treated with LPS+DMSO); the anti-inflammatory cytokine Il10 showed an increasing trend that did not reach statistical significance, whereas Il6 and Tnfα did not show altered levels in mouse sera (Fig. 7C). A Cxcl10 decrease was also confirmed by ELISAs in different mouse cohorts (Fig. 7D). TMs showed similar efficacy *in vivo*, and, notably, this confirmed our *in vitro* results for the inactive acetate prodrug TM8n to be converted, likely via esterases, to the TM7nred/TM7nox redox couple *in vivo* (Fig. 7A). Finally, we observed that TMs behaved differently from dexamethasone in modulating LPS-induced response, as they were unable to modulate Tnfα levels and were equally effective in reducing Il1b, but were more potent in reducing Cxcl10 and increasing Il10, showing *in vivo* efficacy in this model.

## DISCUSSION

After rationally designing four prospective TMs (TM6n, the redox couple TM7nox/TM7nred, and the putative prodrug diacetate TM8n) targeted towards stronger potency on HuR and better bioavailability, we synthesized and submitted them to a preliminary *in vitro* efficacy and physicochemical characterization. For the former, we confirmed HuR targeting for TM6n and TM7nox in cell-free and cellular assays; we observed the lack of cell-free activity for diacetate TM8n, which, conversely, showed cellular activity in line with easy interconversion of the redox couple TM7nox/TM7nred and a putative esterase-activated prodrug mechanism. Computational studies confirmed good affinity for HuR for oxidized ortho-quinones TM6n and TM7nox, while both diphenolic TM7nred and diacetate TM8n were predicted to be inactive. In analytical studies, we confirmed the interconversion of the redox couple TM7nox/TM7nred.

A poor solubility was earlier reported for TMs (Manzoni et al., 2018) and other HuR inhibitors (Lal et al., 2017; Meisner et al., 2007); nevertheless, a careful evaluation of the overall efficacy–stability–bioavailability profiles of our new TMs prompted us to run *in vivo* experiments for the TM7nox/TM7nred redox couple and putative prodrug diacetate TM8n. TMs showed similar efficacy in terms of biochemical activity and cell viability; we selected the ortho-quinone TM7nox as a preferred tool for a detailed mechanistic characterization in terms of putative therapeutic effects on inflammation, keeping in mind its cellular/*in vivo* presence when reduced TM7nred was used. Indeed, we showed the overlapping biological activity of the two species of the redox couple. We investigated the ability of TM7nox to modify the innate immune response triggered by LPS in macrophages. The most relevant mediator of LPS stimuli is the transcriptional activity of the LPS/TRL4/NF-κB axis. However, TMs blunt, but not abolish, such LPS-induced response without inhibiting the primary transcriptional response induced by NF-κB, as demonstrated by our genome-wide data. In addition, other RBPs, such as TTP, TIA1 cytotoxic granule-associated RNA-binding protein-like 1 (TIAR) and heterogeneous nuclear ribonucleoprotein K (HNRNPK), are involved in the post-transcriptional response to LPS in macrophages (Ostareck and Ostareck-Lederer, 2019). In BMDMs, PAR-CLIP and RNA-seq analysis of the response to LPS indicated that HuR and TTP bind to specific target genes, but also compete for a small subset among them containing the binding motifs for each protein (Sedlyarov et al., 2016; Tiedje et al., 2012). Therefore, it is plausible that the sole inhibition of HuR does not completely abrogate the LPS-induced response. Furthermore, our RIP-seq experiments identified the most stably bound transcripts more likely engaged for translation, but, contrary to UV-crosslinked methods, did not provide information about transient, low-affinity interactions between HuR and a specific transcript (Simone and Keene, 2013).

The modulation by TMs results, at least in part, from their ability to interfere with several HuR–AU-enriched target mRNA interactions. We observed a comprehensive remodulation of the transcripts bound by HuR, as, after TM7nox treatment, those that enriched their binding to HuR contain longer 3′-UTR and a higher number of AU/U-rich regions compared to the ones that lost or did not change their binding. This mechanism of action is similar to that observed using DHTS in a different cellular model (Lal et al., 2017). TM treatment indeed decreases HuR binding to mRNAs of major players of the immune response, such as *Cxcl10* and *Il1b*, leading to decreased secretion of the encoded proteins. CXCL10, also known as interferon γ-induced protein 10 (IP-10), is strongly induced by IFNs (IFN-α, IFN-β but mostly IFN-γ) (Karin, 2020; Tannenbaum et al., 1998), as well as by the LPS/NF-κB axis (Ciesielski et al., 2002; Qian et al., 2007) and other immune stimulants in monocytes, endothelial cells, fibroblasts and cancer cells (Tokunaga et al., 2018). By interacting with the chemokine receptor Cxcr3, Cxcl10 stimulates differentiation of naive T cells to T helper 1 (Th1) cells and induces migration of immune cells to site of inflammation (Wildbaum et al., 2002). According to this, the inhibition of CXCL10 is considered beneficial in treating T-cell-mediated autoimmune diseases (Karin, 2020) such as rheumatoid arthritis (Kim et al., 2014; Yellin et al., 2012) and type I diabetes (Frigerio et al., 2002). IL1β belongs to the interleukin-1 family (Dinarello et al., 2010) and is considered a potent pro-inflammatory cytokine mostly linked to the innate immune response (Kaneko et al., 2019). It is a clinical target in autoinflammatory diseases such as cryopyrin-associated periodic syndromes or autoimmune disease (Church and McDermott, 2010). Our data have been collected by co-administering LPS with TM7nox. In so doing, as highlighted in the ActD chase experiments, we observed that TMs modulate the transcription of a core set of LPS target genes by interfering with HuR nuclear activity. The same experiments, in which TMs are administered after LPS stimulus, suggest also a post-transcriptional mechanism that, for the genes investigated, leads to the stabilization of the genes of interest. Further studies are required to investigate this aspect. These limitations notwithstanding, our observations suggest the putative usefulness of TMs in preclinical models of autoinflammatory and autoimmune diseases. A similar effect has been reported by the small-molecule HuR modulators DHTS (Lal et al., 2017) and SRI-42127 (Chellappan et al., 2022). TM7nox disrupts the interaction between HuR and its target mRNAs similarly to DHTS, and the latter was shown to exhibit a strong anti-inflammatory activity by suppressing mRNA expression or secreted protein levels of the pro-inflammatory mediators Tnfα and Il6 in LPS-induced RAW 264.7 cells and BMDMs, as well as *in vivo* in LPS-induced mouse models (Guo et al., 2018; Yue et al., 2022; Gao et al., 2018; Yue et al., 2021). The anti-inflammatory activity of DHTS has also been associated with its ability to suppress nuclear translocation of NF-κB (Yuan et al. (2019), which was not observed in the case of TMs, suggesting a partially different mechanism of action between the two classes of small molecules. In LPS-activated primary microglial cells, SRI-42127 reduces the expression and release of several cytokines and chemokines such as Il1β, Ifn-γ, Ccxl1, Ccl3 and Il6, but has an opposite effect on Cxcl10 (Chellappan et al., 2022). Notably, the spectrum of affected target genes is largely overlapping with the ones affected by TMs, although the mechanism of HuR interaction is different for the two chemotypes. In fact, SRI-42127 is an inhibitor of HuR homodimerization and a blocker of HuR nucleo-cytoplasmic shuttling, which abolishes the cytoplasmic function of HuR, reducing the stability of targeted mRNAs (Chellappan et al., 2022); conversely, TMs, and in particular ortho-quinone TM7nox presented here, bind to HuR between the first two RRMs, competing with target mRNAs and locking the protein in a closed conformation, with no apparent influence on dimerization and nucleo-cytoplasmic shuttling. As shown by our molecular dynamic simulations, TM7nox not only binds between the RRM1-2 domains, but also induces the surrounding loops of the RNA binding cavity to fold around the ligand to dramatically reduce the available buried surface area between the two RRM domains. Thus, such conformational state surely hampers the accommodation of the binding mRNA.

TMs, as shown by RNA-seq and RIP-seq experiments, change the HuR-bound transcriptome and modulate the activity of HuR. As functional data reported here suggest that TMs have a detectable effect while a strong HuR activation takes place, such as after LPS exposure, their putative usefulness during different stress stimuli that lead to HuR activation should be investigated.

## MATERIALS AND METHODS

### Chemical synthesis

Synthetic procedures and compound characterization are reported in the Supplementary Materials and Methods.

### HTRF

A recombinant M1M2 version of HuR was expressed in *E. coli* BL21 cells, as previously described (Lal et al., 2017; D'Agostino et al., 2013, 2015). HTRF was used to determine the inhibition of the binding between His-tag M1M2 HuR recombinant protein and a 26 biotinylated TNF ARE RNA probe (5′-AUUAUUUAUUAUUUAUUUAUUAUUUA-3′) by TMs. We calculated the protein Hook point, by testing different concentrations of the protein (0, 5, 10, 20, 50, 100, 200 and 500 nM) in the presence of an ARE probe (50 nM). After inhibition, tests were performed with the HuR construct (20 nM) and the probe (50 nM), and HTRF experiments were performed according to the manufacturer’s instructions (Cisbio). Signals were measured with a Tecan Spark microplate reader, using the protocol indicated by the manufacturer (Cisbio).

### REMSAs

M1M2 HuR recombinant protein was purified and REMSAs were performed as previously described (Lal et al., 2017; D'Agostino et al., 2013, 2015). Briefly, the protein (3.7 nM) was incubated for 30 min with 5′-DY681-labeled AU-rich RNA probe (1 nM) and DMSO as control, or with TMs at various dosages. Afterwards, samples were loaded on 4% native polyacrylamide gel; images were developed with a Typhoon Trio scanner (GE Healthcare) at the resolution for the DY681-probe.

### Cell line primary cultures

Murine macrophage RAW 264.7 (Interlab Cell Line Collection, Genova, Italy) cell lines were maintained in high-glucose Dulbecco's modified Eagle medium (DMEM) by adding 10% fetal bovine serum (FBS; Lonza), 2 mM L-glutamine, 100 U/ml penicillin-streptomycin (Lonza) in standard growth conditions. Murine BMDMs were obtained from sex-balanced C57BL6/j 6- to 12-week-old mice according to published protocols (Kontoyiannis et al., 1999; Warren and Vogel, 1985). To stimulate differentiation, BMDMs were cultured for 7 days with 10% supernatant medium from L929 fibroblasts (Sigma-Aldrich, 85011425), and maintained in Roswell Park Memorial Institute (RPMI) 1640 medium by adding 5% FBS (Lonza), 2 mM L-glutamine and 100 U/ml penicillin-streptomycin (Lonza) in standard growth conditions (Kontoyiannis et al., 1999; Warren and Vogel, 1985).

### Animal inflammation models and sera collection

C57BL6/j mice were purchased from Charles River Laboratories, bred and maintained in the animal facilities of the Department of Cellular, Computational and Integrative Biology (CIBIO) under pathogen-free conditions and according to the authorization received from the Italian Health Ministry ethical committee for animal experimentation (#629-2018). To measure inflammatory factor secretion, 8-week-old C57BL6/j mice were injected i.p. with LPS (Sigma-Aldrich, L3755) at 150 μg/25 g body weight. Dexamethasone (10 mg/kg) and TMs (40 mg/kg) were co-administrated with LPS via i.p. injection in a solution containing 20% Kolliphor EL and 5% DMSO in PBS. Blood samples were collected 90 min later by cardiac puncture, and two serial 10 min centrifugations, at 850 ***g*** at 4°C and at 3500 ***g*** at 4°C, were performed to collect sera.

### Biotinylated RNA pull-down assay

RAW 264.7 cells (5-10 million) were seeded and treated with either DMSO or TMs (10 µM) for 6 h, then lysed in polysome extraction buffer [20 mM Tris-HCl (pH 7.5), 100 mM KCl, 5 mM MgCl_2_ and 0.5% NP-40 plus RNAse and protease inhibitors], and incubated for 1 h at 4°C with 0.5 μM of positive (Bi-TNF) or negative biotinylated probe (D'Agostino et al., 2013) (Bi-TNFneg, 5′-ACCACCCACCACCCACCCACCACCCA-3′) RNA in TENT buffer [20 mM Tris-HCl (pH 8.0), 2 mM EDTA (pH 8.0), 500 mM NaCl 1% (v/v), Triton X-100] plus 100 units of RNAse inhibitors (Thermo Fisher Scientific) and protease inhibitors (Sigma-Aldrich) (Panda et al., 2016). Solutions were incubated for a further 2 h with 30 μl/samples of streptavidin magnetic beads (Life Technologies, 11205D). Ten percent of the total lysates for each sample was kept and used as input material for subsequent precipitation assay. Specific protein enrichments in bead-precipitated samples were analyzed by immunoblotting and densitometric analysis obtained using ImageJ 1.4 software (National Institutes of Health). Samples were diluted in Laemmli Buffer (6×), denatured at 98°C for 5 min, and then separated by SDS–PAGE and blotted onto PVDF membranes (Immobilon-P, Millipore). Membranes were incubated for 1 h at RT or overnight at 4°C with the following antibodies: mouse anti-HuR (Santa Cruz Biotechnology, sc-71290; dilution, 1:1000; initial concentration, 0.5 mg/ml) and mouse anti-β-actin antibody (Cell Signaling Technology, 3700; dilution, 1:1000; initial concentration, 0.5 mg/ml). Secondary antibodies (Santa Cruz Biotechnology) were used for protein detection, using an ECL (Enhanced ChemiLuminescence) Select Western Blotting Detection Reagent (GE Healthcare, RPN2235). Immunoblotting for β-actin was performed as for control.

### ELISAs and Bio-Plex assays

ELISAs were carried out on mouse sera, and RAW 264.7 cell and BMDM supernatants with several dilutions according to each targeted cytokine. For Cxcl10 detection, RAW 264.7 cell and BMDM supernatants were diluted 1:5. Mouse sera were diluted 1:10 (Cxcl10 detection) and 1:5 (Tnfα detection), according to the manufacturer's instructions (R&D Systems, Mouse CXCL10/IP-10/CRG-2 DuoSet ELISA #DY466 and Mouse TNF-alpha DuoSet ELISA #DY410). Signals were detected using TMB solution (Thermo Fisher Scientific) as a substrate. The reaction was then stopped with 2N H_2_SO_4_ and read with a Tecan microplate reader at 450 nm. Cytokine analysis was performed with the Bio-Plex technology (Bio-Rad Laboratories, Hercules, CA, USA), which combines a sandwich immunoassay with fluorescent bead-based technology, allowing individual and multiplex analysis of up to 100 analytes in a single microtiter well (Vignali, 2000). The assay for mouse Tnfα, Il1b, Il6, Il10 and Cxcl10 (Merck, Darmstadt, Germany) was carried out at Bioclarma, Torino, Italy. Briefly, serum samples were diluted 1:2 in assay buffer and analyzed in 96-well microplates, according to the recommendations of the manufacturer (Bio-Rad Laboratories). The content of each well was then drawn up into the Bio-Plex 100 System array reader (Bio-Rad Laboratories), which identifies and quantifies each specific reaction based on bead color and fluorescent signal intensity. The data were finally processed using Bio-Plex Manager software (version 6.1) using five-parametric curve fitting and converted into pg/ml.

### Immunofluorescence

RAW 264.7 cells (2×10^4^/well) were seeded in a 96-well cell carrier plate, and, after treatment (see Results), they were fixed with 3.7% paraformaldehyde (PFA) for 15 min at RT. Cells were permeabilized for 10 min with permeabilization buffer (0.2% Triton X-100 in PBS) and incubated with blocking solution [2% bovine serum albumin (BSA) in PBS] for 15 min. Primary antibody anti-HuR (1:250 in 3% BSA), anti-NF-κB (1:250 in 3% BSA) and secondary fluorophore-conjugated (Alexa Fluor 594 Red or Alexa Fluor 488) antibody (1:500) were diluted in PBS+0.6% BSA. DAPI (1.5 μg/ml) in PBS+BSA 0.6% was used to detect nuclei. Fluorescence images were acquired using an ImageXpress Micro Confocal (Molecular Devices). In each well, images were acquired in five preselected fields of view with a 20× Plan Apo objective (0.75 NA) over three channels: blue [fluorescence excitation (λEx), 377/54 nm; fluorescence emission (λEm), 432/36 nm]; green (λEx, 475/28 nm; λEm, 536/40 nm); far red (λEx, 635/22 nm; λEm, 692/40 nm). For optimal detection, four *z*-stacks were acquired with a step size of 3 µm in spinning disk confocal mode (60 µm pinhole); the resulting maximum-projection images were used for the analysis. In brief, individual cell nucleus and cytoplasm were segmented using MetaXpress Custom Module Editor (MD), the ratio between nuclear and cytoplasmic signals was calculated for each cell, and the mean value of the well was reported.

### ActD chase experiments

Transcription was blocked with ActD administration at 2.5 µM for 1 h and 3 h to measure mRNA stability. After experimental optimizations, ActD treatments were performed after 3 h stimulation with LPS to guarantee inflammatory response activation. TM7nox (10 µM) was added simultaneously with ActD or with LPS (1 µg/ml) to evaluate its capability to modulate targets transcription or degradation/stability. Total RNA from each sample was extracted with TRIzol, and qRT-PCR was performed to quantify mRNA levels as described in the ‘Total RNA extraction and qRT-PCR’ section.

### Computational details: docking calculations

The crystal structure of the RRM1 and RRM2 HuR domains complexed with mRNA (PDB ID: 4ED5) (Wang et al., 2013) was chosen as starting receptor conformation for docking simulations. The choice of the ‘closed’ form, owing to the presence of mRNA, was based on the capability of our TMs to bind HuR in the mRNA binding region. Both HuR and the ligands were prepared with Maestro Version 12.7.156, the interface for Schrödinger's molecular modeling platform. In particular, the protein was prepared with Protein Preparation Wizard (Sastry et al., 2013), included in Maestro. Hydrogens were added to the protein, and missing side chains were added using the Prime (Jacobson et al., 2004, 2002) module of Maestro. Crystallographic water molecules and the native ligand mRNA were deleted. The N-terminal and C-terminal residues were capped with acetyl (ACE) and N-methyl amide (NME) groups, respectively. To properly describe the protonation state of the protein residues and also correctly describe the hydrogen bonding networks at pH 7, protonation states were assigned by evaluating their pKa with the Propka (Olsson et al., 2011) program included in Maestro. Finally, a relaxation procedure was performed by running a restrained minimization only on initially added hydrogen atoms, according to the OPLS2005 (Banks et al., 2005) force field. TM ligands were generated and then prepared through the LigPrep module of Maestro, employing the OPLS2005 force field. The Epik (Greenwood et al., 2010; Shelley et al., 2007) module of Maestro was used to evaluate the pKa of each ligand at pH 7, to properly describe its protonation state. Each obtained TM ligand was further optimized at molecular mechanics level through the MacroModel program included in the Schrödinger suite of programs. Docking simulations were performed with AutoDock4.2 (Morris et al., 2009). The grid and setting to run docking calculations were prepared by using AutoDockTools, the graphical interface of AutoDock. The grid for docking calculations was computed by using 108×108×68 points, spaced by 0.375 Å. These parameters correspond to a grid of ∼40×∼40×∼25 Å in a 3D Cartesian coordinate system. In so doing, the whole region between the two HuR domains in the *xy* plane was properly included. A hundred independent runs of the Lamarckian genetic algorithm local search method per docking calculation were performed, and a threshold of maximum 25 million energy evaluations per run was applied. Docking conformations were clustered on the basis of their RMSD (tolerance, 2 Å).

### MD simulations

The force field ff14SB (Maier et al., 2015) was used to model HuR. Regarding TM7nox, the generalized Amber force field (Wang et al., 2004) was employed. Restrained electrostatic potential (RESP; Bayly et al., 1993) charges were obtained by using the antechamber accessory module of AmberTools. The electrostatic potential (ESP; Singh and Kollman, 1984) charges employed to calculate RESP ones were evaluated at *ab initio* theory level with the Gaussian software. The TM was optimized by using the density functional theory method that has accurately simulated molecular structural and spectroscopic properties (Donati et al., 2018, 2020; Chiariello et al., 2020; Wildman et al., 2019; Raucci et al., 2020; Battista et al., 2018). In particular, the B3LYP (Becke, 1993)/6-31G* theory level was employed, and then ESP charges were calculated on the optimized minimum energy structure at HF (Roothaan, 1951)/6-31G* theory level. By using the leap program available in AmberTools, a solvent box of 12 Å between any protein atom and the edge of the box was added, where water molecules were described through the TIP3P (Jorgensen et al., 1983) force field. A box of ∼70×∼65×∼85 Å was obtained, and neutrality was ensured by adding six Cl^−^ ions, modeled with Joung and Cheatham (2008) parameters. Finally, coordinates and topology files for the whole system were obtained. Energy minimizations and MD simulations were performed with Gromacs (Bekker et al., 1993; Abraham et al., 2015) software. For all simulations (both equilibration and production runs), the Verlet cut-off scheme was used for non-bond interactions neighbor search. The fast smooth particle-mesh Ewald (Essmann et al., 1995) (SPME) method was employed for long-range electrostatic interactions; the cut-off was set to 1.2 nm for long-range Van der Waals interactions, with the Lennard-Jones potential gradually switching to zero between 1 nm and 1.2 nm. For all MD simulations, the leap-frog (Hockney et al., 1974) algorithm for integrating Newton's equations of motion was used, and a time step of 2 fs was chosen. The equilibration procedure started with two energy minimization steps performed with the steepest descendent algorithm. The first one was 20,000 steps long, and the ligand and protein heavy atoms were kept fixed by imposing a harmonic constraint of 500 kcal mol^−1^ Å^−2^, so that only the solvent was allowed to relax. In a second, 10,000 steps-long equilibration run, the entire system was not constrained. Then, the system was gradually heated by increasing the temperature by 50 K in each step, with subsequent MD runs in the canonical ensemble (NVT), until reaching a final temperature of 300 K. All these steps were 200 ps long, and harmonic restraints were gradually decreased in each step. Beside the first one, constraints of 30 kcal mol^−1^ Å^−2^ were applied on all the heavy atoms of the protein and the ligand; for all the other NVT simulations, constraints of 25, 18, 12, 6.5 kcal mol^−1^ Å^−2^ were applied only on the protein backbone atoms (Ca, N, C, O) and the ligand heavy atoms. During the last NVT step, a constraint of 1.5 kcal mol^−1^ Å^−2^ was applied only on the ligand. The weak-coupling Berendsen (Berendsen et al., 1984) scheme was used for temperature coupling. A final NPT run of 1 ns was performed, without constraints, to adjust the box volume, and the Berendsen algorithm was used for pressure coupling. MD production runs of 2 µs with a time step of 2 fs were performed for each of the chosen poses. For production runs, temperature and pressure controls were carried out with the velocity rescale (Bussi et al., 2007) and Parrinello–Rhaman (Parrinello and Rahman, 1981) scheme, respectively. The LINCS (Hess et al., 1997) algorithm was employed to constrain bonds. Trajectory visualization and analyses were performed with the VMD (Humphrey et al., 1996) software, and figures were obtained using the Pymol molecular visualization system.

### RNA immunoprecipitation and next-generation sequencing (NGS)

RAW 264.7 cells (30 million) were used in RIP experiments, followed by qRT-PCR or NGS sequencing. In general, RIPs were performed without cross-linking steps (Keene et al., 2006), using 1-15 μg/ml anti-HuR antibody (Santa Cruz Biotechnology, sc-71290) and the same amount of mouse normal IgG isotype (negative control; Santa Cruz Biotechnology, sc-2025). Cells were harvested after treatment for 6 h with LPS (1 µg/ml), TMs (10 µM) or DMSO, then lysed with 20 mM Tris-HCl at pH 7.5, 100 mM KCl, 5 mM MgCl_2_ and 0.5% NP-40, supplemented with 1% protease inhibitor cocktail (Sigma-Aldrich) and 100 units of RiboLOCK RNase inhibitor (Thermo Fisher Scientific) for 10 min on ice, and centrifuged at 15,000 ***g*** for 10 min at 4°C. Lysates were incubated with Pierce A/G beads (Thermo Fisher Scientific, 88847-88848) for 1 h at 4°C for pre-clearing steps, and in parallel 80% A and 20% G beads for each sample were incubated with HuR or IgG antibodies (8 μg) for the antibody-coating step for 1 h at RT. Then, lysates were incubated with antibodies and beads for a further 4 h at 4°C. Finally, samples were washed (six times, 5 min each wash) with NT2 buffer. The TRIzol reagent was added directly to the beads for HuR-bound RNA isolation, and they were processed for qRT-PCR analysis or library preparation; 1-5% of the total lysate for each sample was stored as input. For validation experiments, quantitative PCRs were performed after cDNA synthesis (Thermo Fisher Scientific, K1612) using Universal SYBR Master Mix (KAPA Biosystems, KR0389) on CFX-96/384 thermal cyclers (Bio-Rad Laboratories). Fold enrichment for *Cxcl10*, *Cd40*, *Fas*, *Nos2* and *Il1b* was calculated as previously described (D'Agostino et al., 2015). In detail, we applied the equation 2e−ΔCt, in which ΔCt is expressed as the ratio between target mRNA IP HuR on target mRNA IgG. For each condition, the ΔCt values for HuR and IgG IP samples were calculated in triplicate.

### cDNA library preparation

Quantity and quality of RNA samples (RNA-HuR IP; IgG IP and inputs) were measured using a Qubit™ RNA High Sensitivity (HS), Broad Range (BR) Assay Kit (Thermo Fisher Scientific). RNAs from INPUT and RIP were quality-controlled using Bioanalyzer (Agilent Technologies) and 7 ng of the extracted RNA, with RNA integrity number higher than 9, were used for fragmentation at 94°C for 4 min. RNA libraries were generated using a SMART-Seq Stranded Kit (Takara). The kit incorporates SMART^®^ cDNA synthesis technology (Zhu et al., 2001) and generates Illumina-compatible libraries via PCR amplification, avoiding the need for adapter ligation and preserving the strand orientation of the original RNA. The ribosomal cDNA was depleted by a ZapR-mediated process, in which the library fragments originating from rRNA and mitochondrial rRNA are cleaved by ZapR in the presence of mammalian-specific R-Probes. Library fragments from non-rRNA molecules were enriched via a second round of PCR amplification using Illumina-specific primers. Quantity and quality of each individual library were defined using a Qubit Fluorometer (Thermo Fisher Scientific) LabChip GX (Perkin Elmer). After libraries' equimolar pooling, the final pool was quantified by quantitative PCR (KAPA Biosystems and Bio-Rad Laboratories). The adaptor-tagged pool of libraries was loaded on a NovaSeq6000 SP flowcell (PE100 Chemistry) for cluster generation and deep sequencing, producing an average of 40 M reads per sample (Illumina, San Diego, CA, USA).

### Bioinformatics analysis

RNA-seq and RIP-seq were performed in quadruplicate. Raw sequence file quality was checked via FastQC (v 0.11.9). Gene quantification was conducted with STAR (version 2.7.7a) starting from Ensembl GRCm39 genome version. The generated genes counts were analyzed using DESeq2 package. The normalized count matrix (obtained from variance stabilizing transformation method as implemented in DESeq2 package) was used to explore high-dimensional data property with PCA coupled with a dimensionality reduction algorithm used in the DESeq2 package. DEGs were selected with a *P-*adjusted cut-off of 0.05 and a log2FC value greater than 1 (upregulated DEGs) or lower than −1 (downregulated DEGs). *P-*value was adjusted for multiple testing using the Benjamini–Hochberg (BH) correction with a false discovery rate (FDR)≤0.05. DEGs were then analyzed with a hierarchical clustering method, using correlation distance. Visualization of *z*-score-normalized values and clustering was obtained via pheatmap package; visualization of DEGs in volcano plots was acquired using the EnhancedVolcano package. Venn diagrams were created using vennPlot (systemPipeR package). Bar plots of genes of interest were created using ggbarplot, annotate_figure, ggarrange (ggpubr package), add_pval (ggpval). Functional annotation was performed for all the comparisons and for the gene list of interest. We used both clusterProfiler and Reactome Bioconductor packages, visualizing the results with ggplot2 and ggpubr packages. The full enriched annotation table results are provided in Table S2. A tree plot and two different networks of the most important pathways resulting from the functional annotation analyses were generated using the Bioconductor package enrichplot.

The correlation analyses among RNA-seq and RIP-seq shared genes was performed using cor.test function, and, for better visualization, we subtracted the two correlation data and plotted the result in a 3D representation, generated with a Delaunay triangulation and a Dirichlet tessellation. In the analysis of the properties of the 3′-UTRs [the lengths and AU/U-rich regions (ARE)] between the differential genes in the TM7nox and LPS co-treatment versus LPS and DMSO RIP-seq, we also included the not differential genes as a control. The lengths of the 3′-UTRs were calculated from the 3′-UTR sequences downloaded from Ensembl (accessed in August 2022). The ARE was downloaded from the Database for AU-rich elements and direct evidence for interaction and lifetime regulation (AREsite2, accessed in August 2022). These data were visualized via boxplots, and statistical support was provided by a Wilcoxon test. Ten HuR binding sites were obtained from EuRBPDB, a database for eukaryotic RBPs containing 315,000 RBPs from 160 species (Liao et al., 2020).

### Total RNA extraction and qRT-PCR

Total RNA was extracted either with a RNA extraction kit (Zymo Research), according to the manufacturer’s instructions or with TRIzol, chloroform precipitation followed by RNAse free-DNAse I treatment, 15 min at 37°C. cDNA Synthesis (Thermo Fisher Scientific, K1612) was performed from 1 µg RNA, and qRT-PCRs were performed using Universal SYBR Master Mix (KAPA Biosystems, KR0389) on CFX-96/384 thermal cyclers (Bio-Rad Laboratories) (Manzoni et al., 2018; Lal et al., 2017; D'Agostino et al., 2015). Normalized expression levels for each selected gene were calculated as 2e−ΔΔCt, where the Ct value of either control or treatment conditions was subtracted from the Ct value of the housekeeping gene (*Rplp0*) to yield the ΔCt value. Then, the ΔCt values for treatment and control were computed in duplicate and averaged to give one ΔΔCt value per sample.

### Statistical analysis

Statistical analysis experiments were performed in a number of biological replicates indicated in all experiments reported in the Results section. *t*-tests were used to calculate final *P*-values, without assuming variances to be equal (Welch's *t*-test). *P*<0.05 was considered significant.

## Supplementary Material

10.1242/dmm.050120_sup1 Supplementary information
