# Supplementary material for: HuR modulation counteracts lipopolysaccharide response in murine macrophages
Source: Dis Model Mech. 2023 Mar 29;16(3):dmm050120. doi: 10.1242/dmm.050120 (PMC10110401; doi:10.1242/dmm.050120)
Supplement: Supplementary information [file dmm-16-050120-s1.pdf]

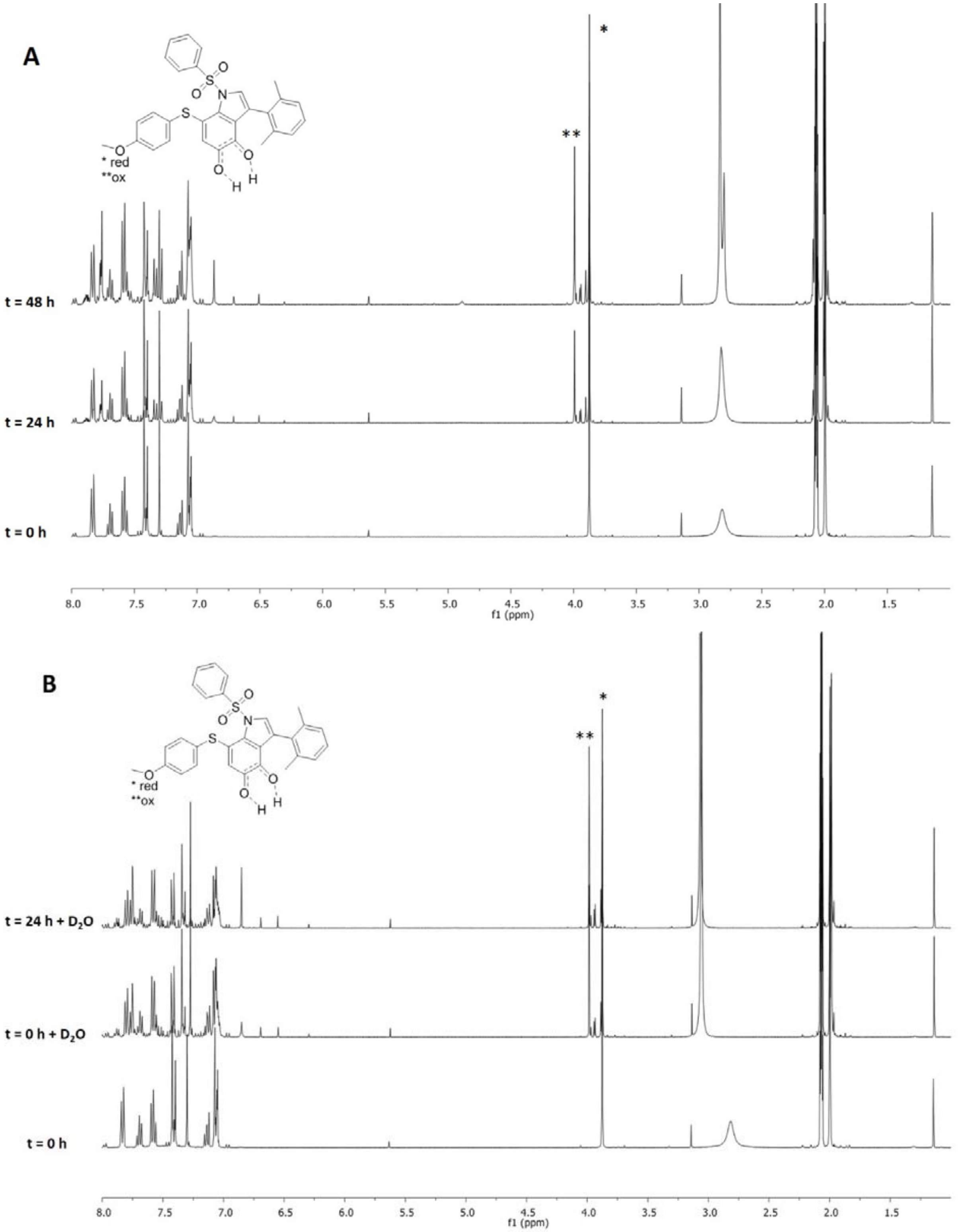

Fig. S1. ^{1}H-NMR studies to evaluate the 4red / TM7nred - 4ox / TM7nox interconversion equilibrium. A) Diphenol **TM7nred** equilibration at room temperature into quinone **TM7nox** up to 48 hours when dissolved in acetone-d_6; B) **TM7nred** equilibration at room temperature into quinone **TM7nox** up to 24 hours after further addition of 10% D_2O.

Disease Models & Mechanisms • Supplementary information

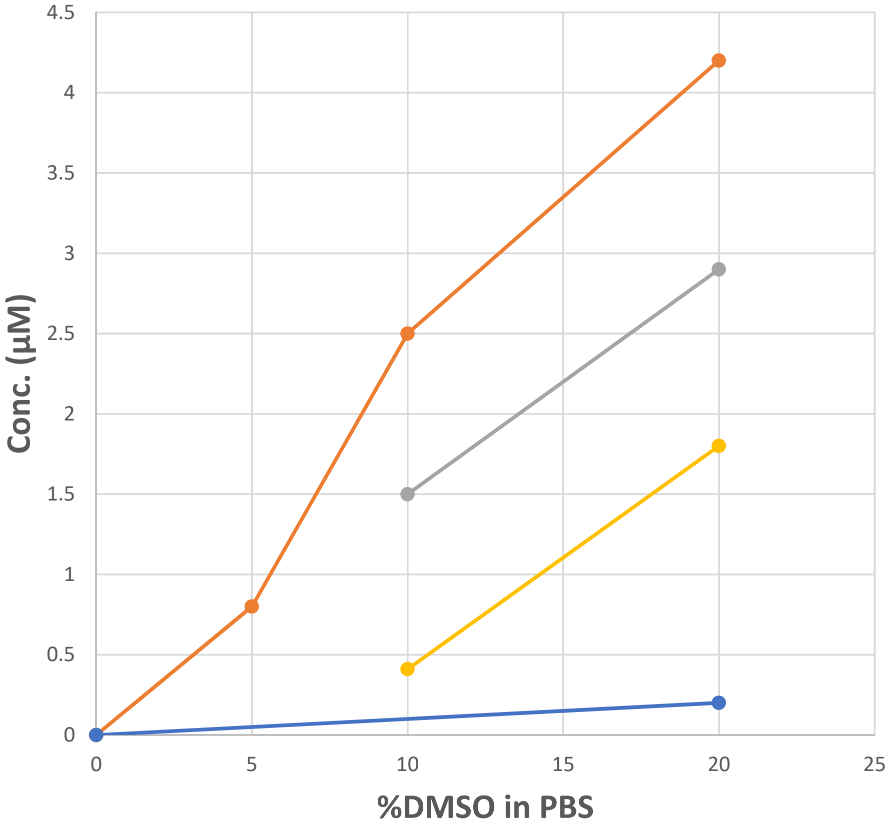

Fig. S2. Graphic summary of kinetic solubility of tanshinone mimics 3 / TM6n (red), 4 r e d /TM7nred (gray), 4ox / TM7nox (yellow) and 5 / TM8n (blue) in PBS containing 20% DMSO.

Disease Models & Mechanisms • Supplementary information

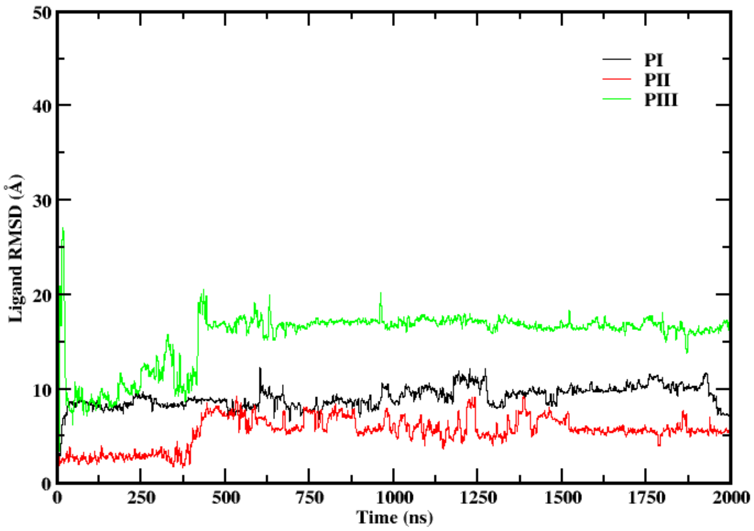

Fig. S3. Ligand RMSD (averaged every 100 steps) temporal evolution for the three poses.

Disease Models & Mechanisms • Supplementary information

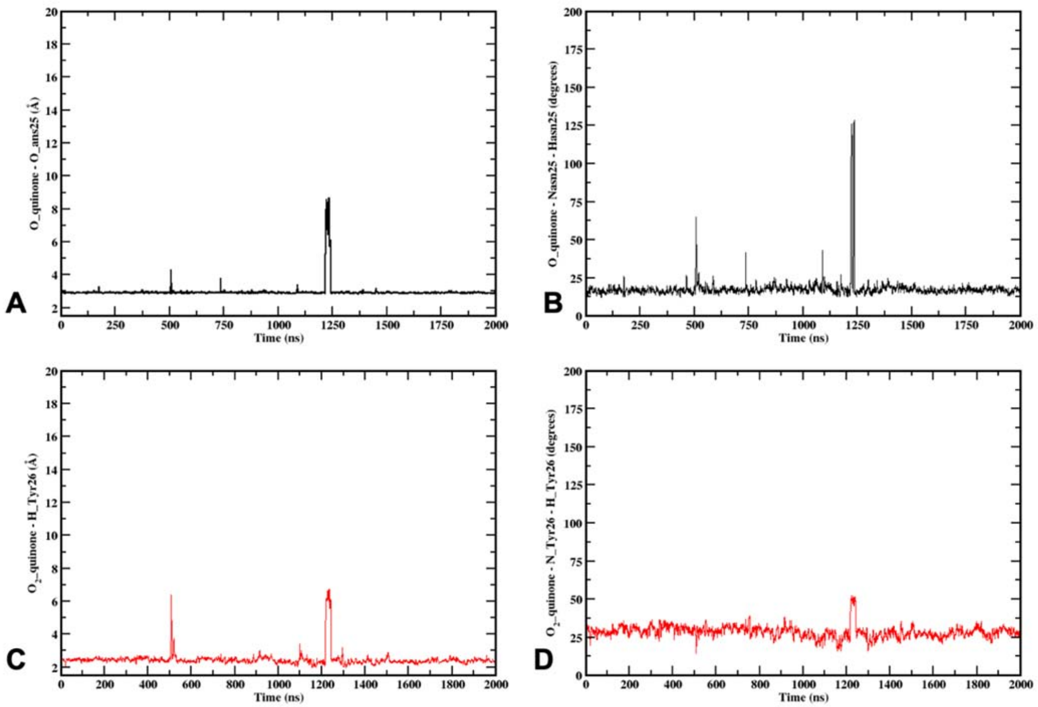

Fig. S4. Hydrogen bond distance and hydrogen bond angle involving quinone oxygens and N25 (A and B) and Y26 (C and D) in the PII trajectory.

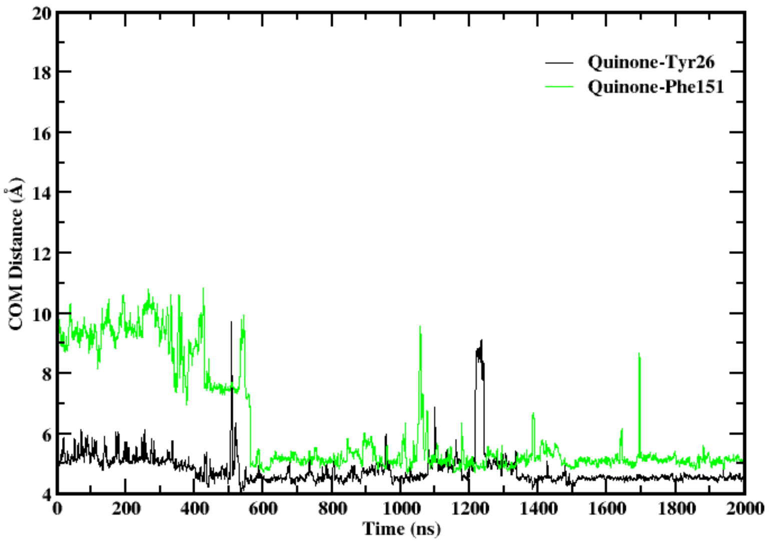

Fig. S5. Distances between the center of mass of the central indole-quinone and Y26 (black line) and F151 (green line) aromatic rings in the PII trajectory. The latter, after the first 500 ns, flips from its original conformation to better interact with **TM7nox**. Only heavy atoms are considered for the center of mass calculations.

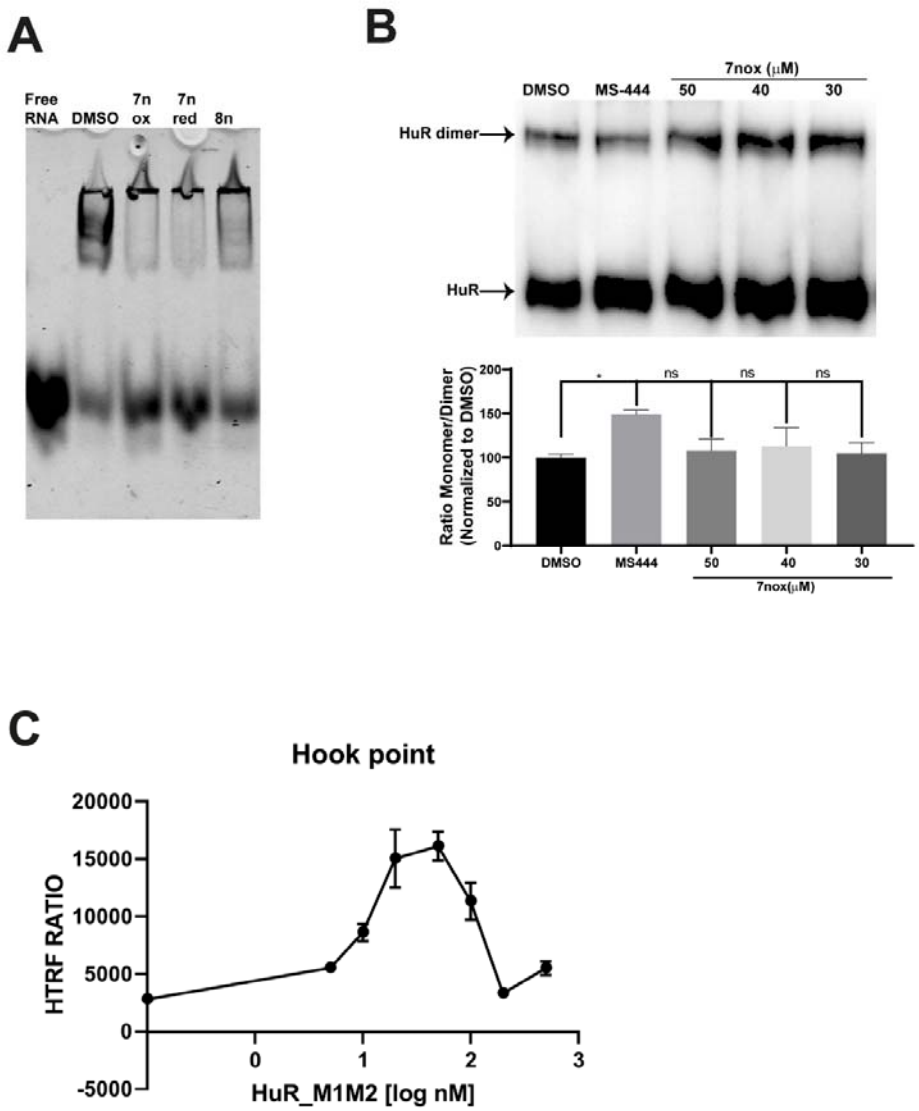

Fig. S6. TMs show inhibitory activity in vitro, except TM-8n. A) Representative REMSA showing HuR-RNA binding impairment induced by **TM7nox** and **TM7nred**, but not **TM8n** (10μM). rM1M2_HuR was incubated for 30 min with either 1 nM of 5′-DY681-labeled RNA probe alone, or together with DMSO used as control and TM **7nox**, **7nred** and **8n**. B) Representative Western Blot (non-reducing and non-denaturing SDS-PAGE) of rM1M2HuR showing bands corresponding to HuR monomeric form (24 kDa) and the dimeric one (48 KDa). With increasing concentrations of **TM7nox** (50, 40, 30 μM) the ratio between the HuR monomer/dimer is not changing respect to control condition (DMSO). MS444 (50 μM) was used as a positive control, since it reduces HuR oligomerization (Meisner et al., 2007) Data plotted as mean ± SD of a biological duplicate (*$p<0.05$). C) Graph showing rM1M2_HuR hookpoint calculation by using HTRF assay. Several quantities of rM1M2 [0, 5 nM, 10 nM, 20 nM, 50 nM, 100 nM, 200 nM, 500 nM] were incubated with 50 nM of 5' biotinylated TMF ARE RNAprobe. Plotted bars are mean ± SD of three independent experiments.

Disease Models & Mechanisms • Supplementary information

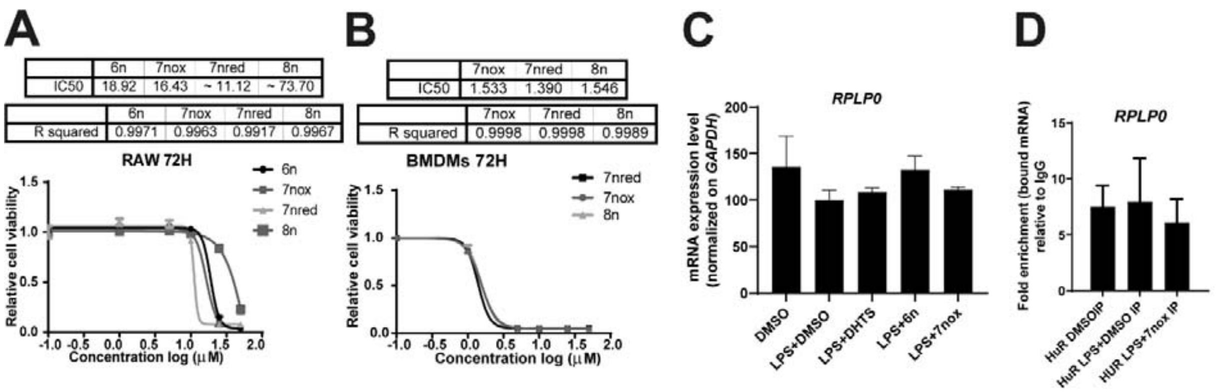

Fig. S7. TMs show toxicity in RAW 263.7 and BMDMs. A) RAW Cell viability was evaluated with OZBlue kit after 72 hours of treatments. Doses were from 0 to 50 µM. Plotted bars are mean ± SE of a biological duplicate, normalized to control (DMSO). Relative IC50 and R2 were calculated by nonlinear regression curve fitting. B) BMDMs viability was evaluated with OZBlue kit after 24 hours of treatments. Doses were ranging from 0 to 50 µM. Plotted bars are mean ± SE three independent experiments performed in technical duplicate, normalized to control (DMSO). Relative IC50 and R2 were calculated by nonlinear regression curve fitting. C) TMs do not modulate mRNA expression level of a non-HuR target. RAW cells were treated for 6 hours with DMSO, LPS+DMSO, LPS+DHTS, LPS+**TM6n** and LPS+**TM7nox** as previously described. qRT-PCRs were performed to evaluate the mRNA level of *RPLP0,* an housekeeping, non-HuR target. Data represent mean ± SD of three independent experiments. D) HuR has been immunoprecipitated in RAW cells after 6 hours treatments with DMSO, LPS+DMSO and LPS+**TM7nox** as previously described. qRT-PCR has been performed to demonstrate that **TM7nox** does not alter the immunoprecipitated level of a non-HuR target, *RPLP0.*

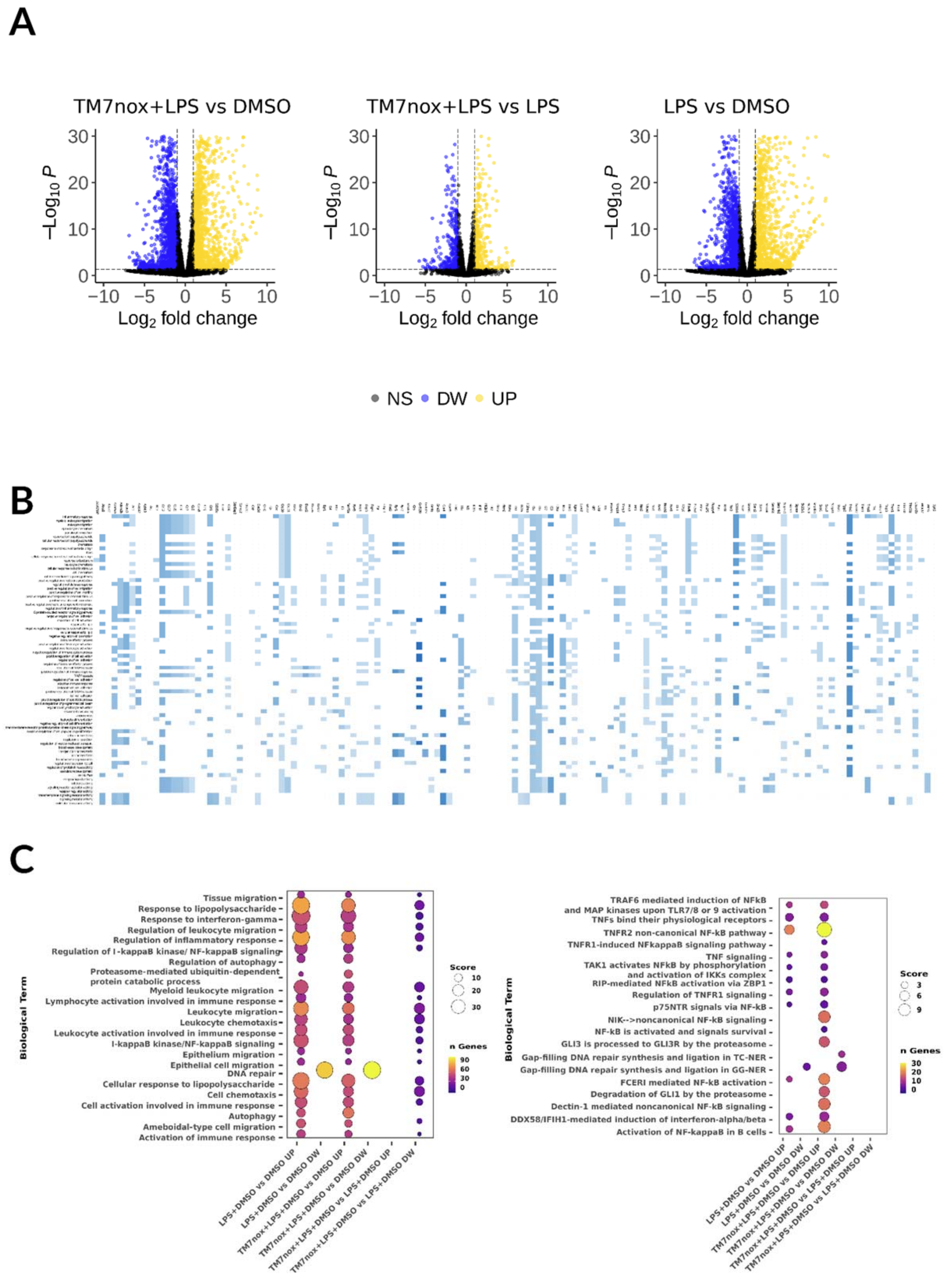

Disease Models & Mechanisms • Supplementary information

Fig. S8. Correlation plots of RNA-seq DEGs and RIP-seq enriched genes. A) Volcano plots of the distribution of DEGs in the three performed comparisons in the RNA-sequencing. Measurement of gene expression fold-change is present on the X-axis against a measure of statistical significance [-Log10 (P-value)] on the Y-axis. Differential genes are established at |Fold change| ≥ 1 and P-value ＜0.05 (yellow: up-regulated DEGs; blue: down-regulated DEGs; grey: no changes in DEGs). B) Heatmap of the 249 down-regulated genes modulated by **TM7nox** in presence of LPS. On y-axis the list of pathways enriched for this subset of genes, on the x-axis the list of the 249 genes. Coloration performed for log_2 FoldChange values. C) Balloon plots of Gene Ontology Term Enrichment and Reactome for pathways of interest across comparisons. Colour of the balloons are illustrated from yellow to purple in descending order of gene counts. Balloons' size is from small to large in ascending order of score.

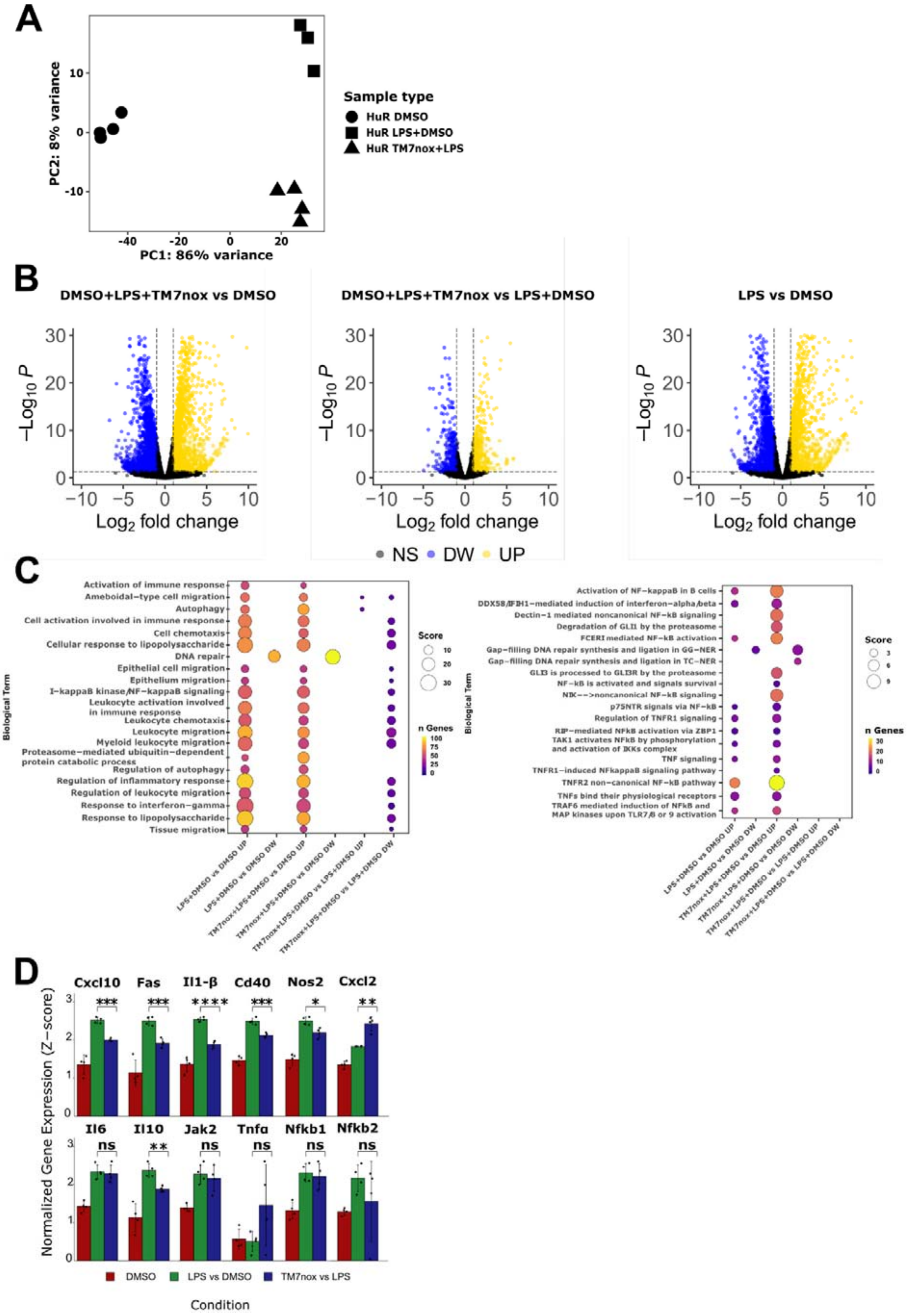

Disease Models & Mechanisms • Supplementary information

Fig. S9. TM7nox modulates HuR bound transcriptome.

A) Principal Component Analysis (PCA) of the 12 samples. PC1 shows 83% variance and . PC2 8% Each dot represents a DMSO sample, each triangle is a sample treated with DMSO + LPS, while each square is a DMSO + LPS + **TM7nox** treated sample. Every condition groups together with the same type of samples and it can be observed that **TM7nox** effect separates DMSO+LPS from DMSO+LPS+**TM7nox**. B) Volcano plots of the distribution of DEGs in the three performed comparisons in the RIP-sequencing. Measurement of gene expression fold-change is present on the X-axis against a measure of statistical significance [-Log10 (P-value)] on the Y-axis. Differential genes are established at |Fold change| ≥ 1 and P-value $<$0.05 (yellow: up-regulated DEGs; blue: down-regulated DEGs; grey: no changes in DEGs). C) Balloon plots of Gene Ontology Term Enrichment and Reactome for pathways of interest across comparisons. The color of the balloons is illustrated from yellow to purple in descending order of gene counts. Balloons' sizes are from small to large in ascending order of score. D) Barplot of Z-score of key genes across different samples (red: DMSO; green: DMSO + LPS; blue: DMSO + LPS + **TM7n**). The significance between DMSO+LPS and DMSO+LPS+**TM7n** samples is shown by an asterisk for each gene and standard error represented by error bars.

Disease Models & Mechanisms • Supplementary information

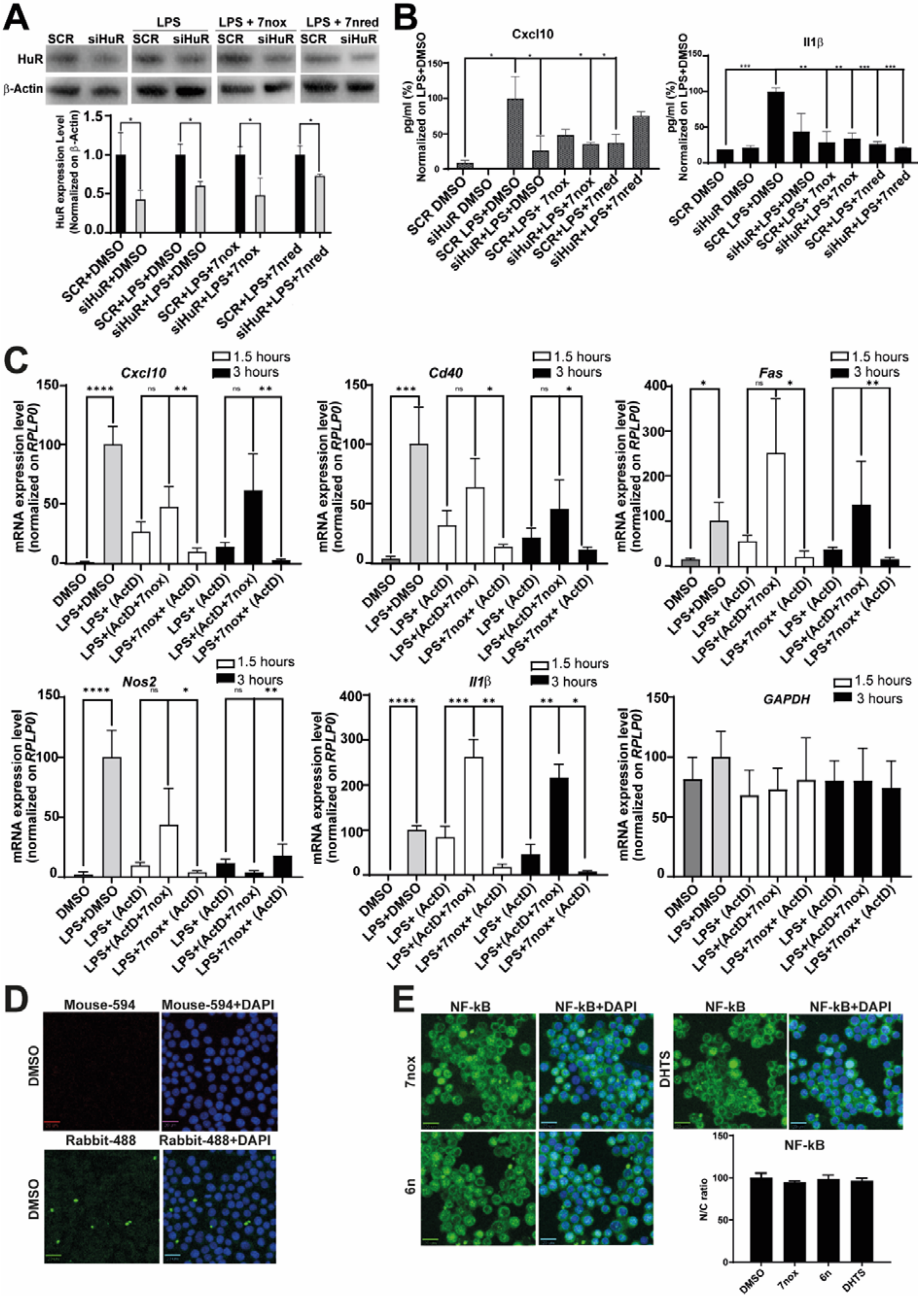

Disease Models & Mechanisms • Supplementary information

Fig. S10. HuR silencing reduces cytokine protein level, TMs treatment increase mRNA stability of selected mRNAs.

A) Representative western blot showing HuR protein silencing with relative quantification. RAW 264.7 cells have been co-treated with DMSO, LPS+DMS0, LPS+**TM7nox** and LPS+**TM7nred** as previously described. Cell lysates cell extracts were boiled and subjected to 15%-SDS-PAGE and resolved proteins were transferred to PVDF membrane (Millipore, IPVH00010). Blocking of the membrane was performed with non-fat dried milk 1% concentration for 1 hour. HuR (Santa Cruz, sc-5261) or b-ACTIN (4967, CST) antibodies were added to the membranes at 1:1000 dilution for overnight staining. Appropriate secondary antibodies (anti-mouse HRP, 115-035-003; anti-rabbit HRP, 111-035-003, Jackson ImmunoResearch) were added for 1 hour staining RT, signals were further revealed with ECL and measured at BioRad ChemiDoc. Quantifications were measured with ImageJ software and Data represented as mean ± SD of a biological triplicate (*p ≤0.05). B) Cxcl10 and Il1b intracellular levels in RAW 264.7 cells after HuR silencing and **TM7nox** and **TM7nred** treatments as previously described. Protein levels were measured with ELISA, RAW 264.7 cells in which HuR was silenced for 48 and cells were treated for further 6 hours with DMSO as control, LPS plus DMSO or TMs. Respectively, 30 and 5 μg of cellular lysates were loaded to measure Cxcl10 and Il1b pg/mL, respectively. After, data have been normalized on LPS+DMSO as control and number are expressed in percentage. Finally, data represent as mean ± SD of a biological triplicate (**p<0.05, **p<0.01, ***p<0.001). C) Actinomycin-D chase experiments to evaluate TM7nox modulatory activity of transcripts stability. RAW cells were co-treated with DMSO, LPS+DMSO, for three hours. Act-D (2.5 mM) was then added and samples were collected after 1.5 or 3 hours. **TM7nox** (10 μM) was administered in combination with LPS or together with ActD at 1.5 and 3 hours to measure its effect on HuR bound mRNAs transcription or stability. Finally, qRT- PCRs were performed to quantify the remaining *Cxcl10*, *Il1*β, *Cd40*, *Fas*, *Nos2* and *Gapdh* mRNA levels. Data are represented as mean ± SD of a biological triplicate (*p ≤0.05, **p ≤0.01, ***p ≤0.001, ****p<0.0001; ns, not significant). D) Representative Immunofluorescence control staining. Control cells were stained with anti-mouse-594 and anti-rabbit 488 antibodies (A- 11032, A-11008) and no backgrounds or non-specific signals were measured. E) Representative immunofluorescence showing no induction of N-kB nuclear translocation inside the nuclei upon treatments with different TMs alone. Cells have been treated for 3 hours with TMs (10 μM).In the graph, the ratio of NFkB fluorescent signal between nucleus and cytoplasm (N/C) is plotted.

Disease Models & Mechanisms • Supplementary information

Table S1. List of Differentially Expressed Genes in all the comparisons. Each sheet reports a different condition; each label is described in the sheet “Data Description.”

Click here to download Table S1

Table S2. Functional Annotation according to Gene Ontology classification of each comparison. Each sheet reports a different condition; each label is described in the sheet “Data Description.”

Click here to download Table S2

Table S3. Functional Annotation according to Gene Ontology classification of specific subset of genes as reported in the text. Each sheet reports a different condition; each label is described in the sheet “Data Description.”

Click here to download Table S3

Table S4. Sequences of the ten HuR binding sites retrieved from Eukaryotic RBP Database.

Click here to download Table S4

Supplementary Materials and Methods

Cell viability assays

Cells were seeded and treated in 96 well-plate for 72 hours and viability was assessed with OZBlue assay. Briefly, OZBlue was added at 10% volume of culture media to each well and cells were further incubated at 37°C, until color signal development was observed. Fluorescence was then measured (excitation 560 and emission 590 nm) with Tecan microplate reader. Cell survival was calculated with respect to control (DMSO), and IC50 values were determined by fitting with GraphPad Prism software v5.1. Crystal violet staining was used to determine cell number for ELISA assay normalization. Cells were fixed in methanol for 15' at RT and then stained with Crystal Violet for 20' (RT). Crystal violet was then washed with water and cells were incubated in 30% acetic acid. Signal was measured as absorbance at 570 nm at Varioskan Lux Reader (Thermo Fisher Scientific).

List of qRT-PCR primers

Cxcl10 For-TGTTGAGATCATTGCCACGA,Rev-GGAGCCCTTTTAGACCTTTTT

Cd40 For-CATCTGTGGTTTAAAGTCCCG, Rev-GGTTTCTTGACCACCTTTTTGAT

Fas For-CCTCCAGTCGTGAAACCATA, Rev-CTATCTTGCCCTCCTTGATGTTA

Nos2 For GAAGTGTCAGTGGCTTCCA, Rev-TCCTTTGAGCCCTTTGTGCT

Il1β For-GCAGCTGGAGAGTGTGGATC, Rev-GCCCATACTTTAGGAAGACAC

HuR For-ATGAAGACCACATGGCCGAAGACT, Rev-AGTTCACAAAGCCATAGCCCAAGC

Rplp0 For-TTTCTGGAGGGTGTCCGCAA, Rev-GCCTTGACCTTTTCAGTAAGT

Chemical Synthesis

General

All chemicals and solvents were of reagent grade and were used without further purification. Solvents were distilled and dried according to standard procedures, and reactions in anhydrous conditions were performed under nitrogen or argon atmosphere. The reactions were monitored by analytical thin-layer chromatography (TLC) using silica gel 60 F_{254} pre-coated glass plates (0.25 mm thickness). Visualization was accomplished by irradiation with a UV lamp and/or staining with Cerium/Molibdate reagent, ninhydrin or permaganate. Purifications were carried out either by

Disease Models & Mechanisms • Supplementary information

flash chromatography on Macherey-Nagel silica gel (particle size 60 µm, 230–400 mesh), or by Biotage™ C18 reverse phase chromatography using KP-C18-HS (35–70 µm) cartridges. NMR spectra were recorded on Bruker AC 300 or Advance 400 instruments in deuterated solvents at 300 or 400 MHz (^{1}H-NMR) or at 75/101 MHz (^{13}C NMR spectra). Chemical shifts δ are expressed in ppm with the solvent reference relative to tetramethylsilane (TMS) employed as the internal standard. Coupling constants are given in Hertz and rounded to the nearest 0.1 Hz; the following abbreviations are used to describe spin multiplicity: s = singlet, d = doublet, t = triplet, q = quartet, m = multiplet, bs = broad signal, dd = doublet of doublet, ddd = doublet of doublet of doublet, ddt = doublet of doublet of triplet, td = triplet of doublet. LC–MS data were collected with a Waters Acquity™ Ultra performance LC equipped with an Acquity UPLC™ HSS T3 column (2.1 mm x 50 mm, 1.8 µm) and a SQD detector.

3-(2,6-dimethylphenyl)-5-methoxy-1-(phenylsulfonl)-1H-indole ***7****.*

Bromomethoxy **6** was synthetized starting from commercial 5-methoxy-1H-indole, following a previously reported procedure[1]. Under argon atmosphere, 2,6-dimethylphenyl boronic acid (28.2 g, 188 mmol, 1.5 eq) and a 5% solution of Na_2CO_3 (26.5 g, 250 mmol, 2 eq) were sequentially added to a vigorously stirred solution of compound **6** (45.8 g, 125 mmol, 1 eq) in dioxane (800 mL). Under stirring, $Pd(PPh_3)_4$ (7.3 g, 6.32 mmol, 0.05 eq) was added to the reaction mixture and the resulting mixture was left stirring for 16 h at 90°C. The reaction mixture was then cooled to rt and filtered through a silica gel pad. The filtrate was concentrated under reduced pressure and treated with Methyl-t-Butyl ether (MTBE). While a solid residue was filtered, the combined organic phases were dried over Na_2SO_4, and concentrated under reduced pressure. The residue was diluted with methanol, the obtained precipitate was filtered, washed with hexane, and dried in vacuum to give 26.7 g of sufficiently pure compound **7** (68.2 mmol, 55% yield).

The following abbreviations are used to describe spin multiplicity: s = singlet, d = doublet, t = triplet, q = quartet, m = multiplet, bs = broad signal, dd = doublet of doublet, ddd = doublet of doublet of doublet, ddt = doublet of doublet of triplet, td = triplet of doublet.

A small amount was further purified by flash chromatography (eluent: 9:1 *n*-hexane/EtOAc) affording pure **7** as a white solid for analytical characterization. MS (ESI^+): m/z 392.33 [$M+H^+$]. Calculated MS for $C_{23}H_{21}NO_3S$: 391.12. ^{1}H NMR (400 MHz, acetone-d6) δ: 8.05 – 7.95 (m, 3H, H7, H8), 7.67 (m, 1H, H10), 7.60 – 7.55 (m, 2H, H9), 7.54 (s, 1H, H1), 7.21 (dd, J = 6.5 Hz, J = 8.5 Hz, 1H, H12), 7.14 (m, 2H, H11), 7.00 (m, 1H, H6), 6.49 (d, J = 2.5 Hz, 1H, H4), 3.68 (s, 3H, OCH_3), 1.94 (s,

Disease Models & Mechanisms • Supplementary information

6H, CH_3). ^{13}C NMR (100.6 MHz, acetone-d6) δ: 157.2, 137.5, 134.1, 132.1, 131.2, 129.9, 129.4, 127.9, 127.3, 126.8, 125.4, 123.2, 115.0, 114.0, 104.6, 102.6, 55.0, 19.6.

^1H NMR (400 MHz, acetone-d6)

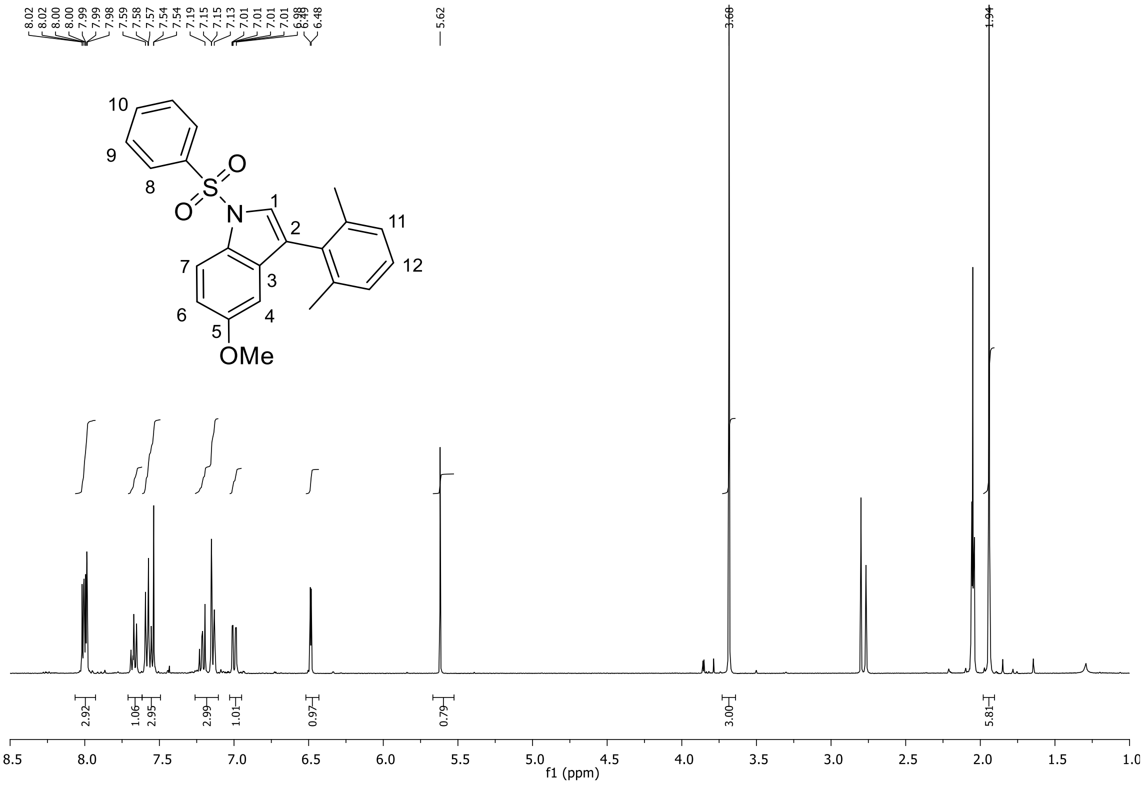

^{13}C NMR (100.6 MHz, acetone-d6)

Disease Models & Mechanisms • Supplementary information

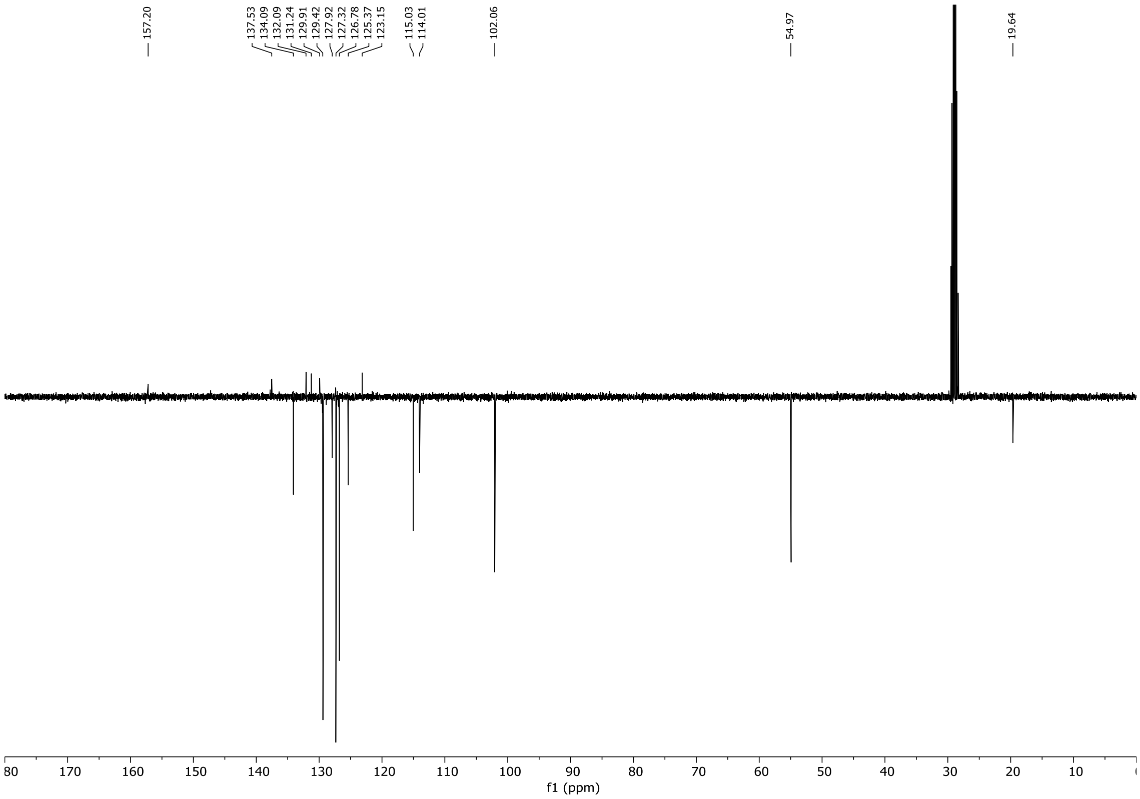

Disease Models & Mechanisms • Supplementary information

3-(2,6-dimethylphenyl)-1-(phenylsulfonyl)-1H-indol-5-ol ***8.***

A solution of BBr_3 (102.7 g, 410 mmol, 6 eq) in DCM (400 mL) was added to a vigorously stirred solution of compound **7** (26.7 g, 68.2 mmol, 1 eq) in DCM (350 mL) at -78°C and under argon atmosphere. After 210 minutes, the reaction mixture was poured into ice, basified with aqueous $NaHCO_3$ to pH=8, and diluted with DCM. The organic layer was washed with water, dried over Na_2SO_4, and concentrated to obtain 23 g of sufficiently pure compound **8** (60.9 mmol, 90% yield). A small amount was further purified by flash chromatography (eluent: 8:2 *n*-hexane/EtOAc) affording pure **8** as a white solid for analytical characterization. MS (ESI$^+$): m/z 378.23 [M+H$^+$]. Calculated MS for $C_{22}H_{19}NO_3S$: 377.11. ^{1}H NMR (400 MHz, acetone-d6) δ: 8.13 (s, 1H, OH), 7.99 – 7.97 (m, 2H, H8), 7.93 (d, J = 8.9 Hz, 1H, H7), 7.69 – 7.64 (m, 1H, H10), 7.59 – 7.55 (m, 2H, H9), 7.50 (s, 1H, H1), 7.20 (dd, J = 6.4 Hz, J = 8.6 Hz, 1H, H12), 7.14 – 7.12 (m, 2H, H11), 6.93 (dd, J = 2.4 Hz, J = 8.9 Hz, 1H, H6), 6.40 (d, J = 2.4 Hz, 1H, H4), 1.93 (s, 6H, CH_3). ^{13}C NMR (100.6 MHz, acetone-d6) δ: 154.5, 137.8, 137.4, 134.0, 132.4, 131.4, 129.4, 129.3, 127.9, 127.3, 126.8, 125.3, 123.0, 115.0, 114.1, 104.6, 104.6, 19.6.

^{1}H NMR (400 MHz, acetone-d6)

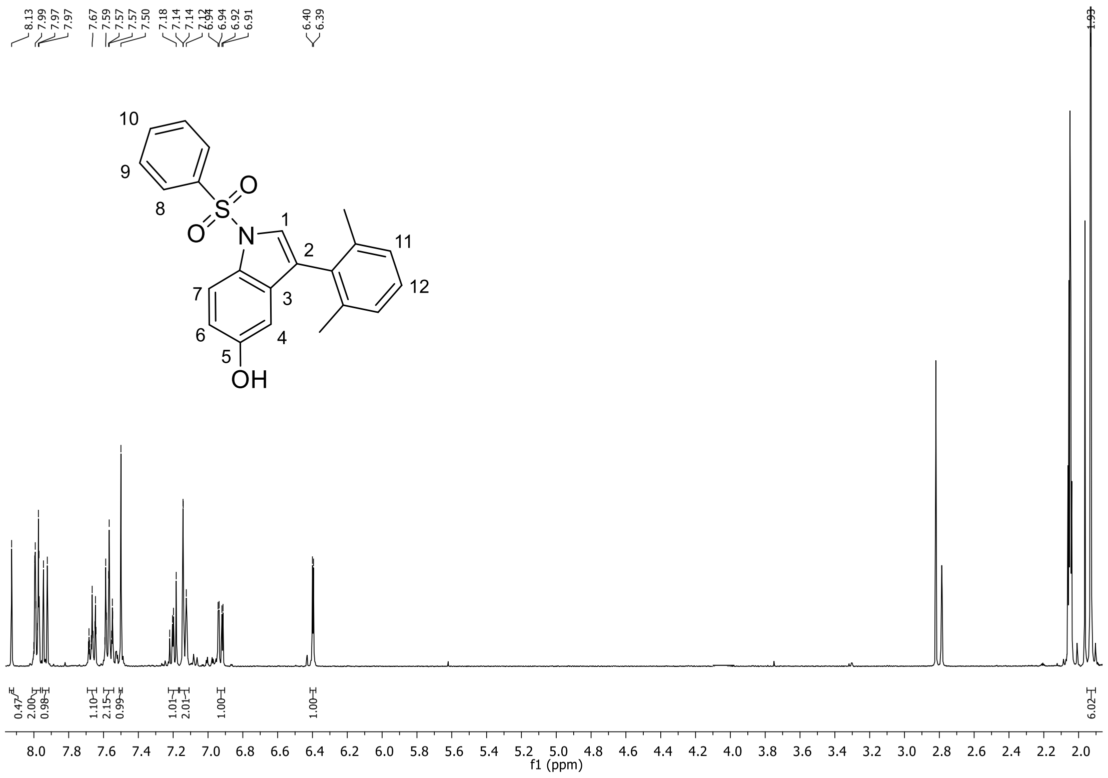

^{13}C NMR (100.6 MHz, acetone-d6)

Disease Models & Mechanisms • Supplementary information

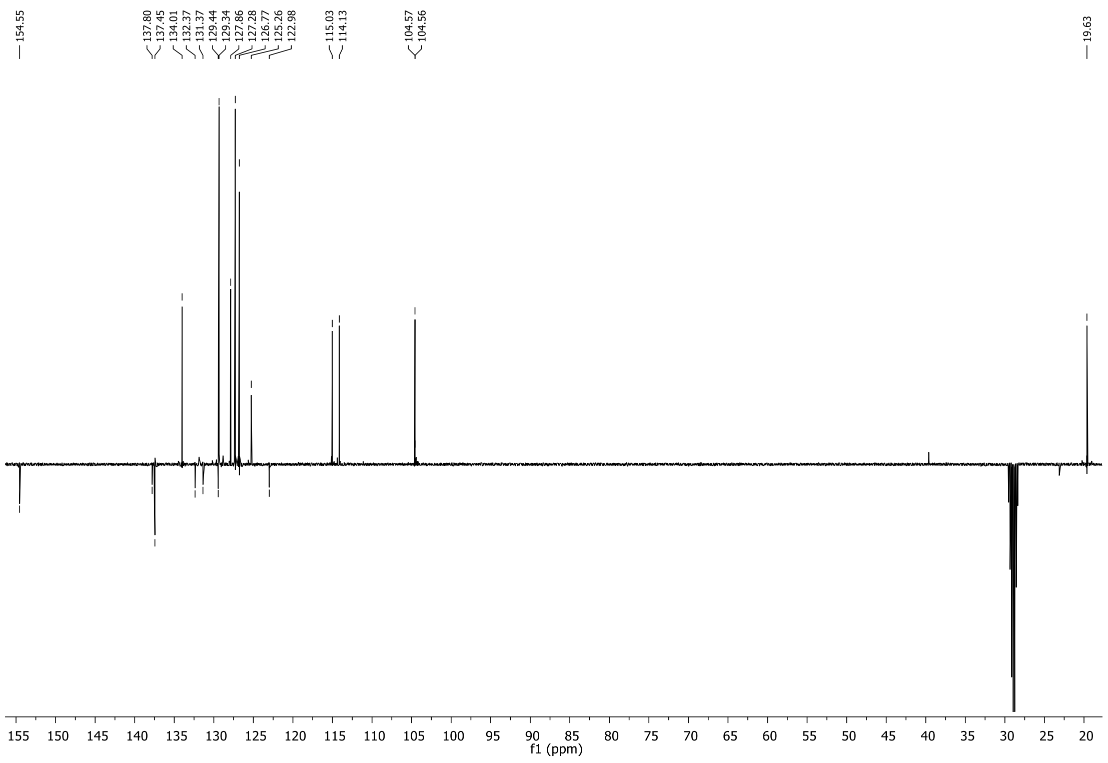

Disease Models & Mechanisms • Supplementary information

*3-(2,6-dimethylphenyl)-1-(phenylsulfonyl)-1H-indole-4,5-dione **9**.*

1-Hydroxy-1,2-benziodoxol-3(1H)-one 1-oxide (IBX, 10.7 g, 38.3 mmol, 1.5 eq) was added in portions to a vigorously stirred solution of compound **8** (9.6 g, 25.4 mmol, 1 eq) in DMF, and the resulting mixture was stirred for 3 h at r.t. Water was added, and the resulting mixture was extracted with EtOAc. The organic layer was washed with 5% aqueous $NaHCO_3$ and water, dried over Na_2SO_4, and concentrated to yield 4.4 g of sufficiently pure target compound **9** (11.2 mmol, 88% yield).

A small amount was further purified by flash chromatography (eluent: from 7:3 to 6:4 *n*-hexane/EtOAc), affording **9** as a red solid for analytical characterization. MS (ESI^+): m/z 392.10 [$M+H^+$]. Calculated MS for $C_{22}H_{17}NO_4S$: 391.09. ^1H NMR (400 MHz, acetone-d6) δ: 8.22 – 8.20 (m, 2H, H8), 8.07 (d, J = 10.5 Hz, 1H, H7), 7.91 – 7.86 (m, 1H, H10), 7.80 – 7.76 (m, 2H, H9), 7.47 (s, 1H, H1), 7.14 (dd, J = 6.7 Hz, J = 8.3 Hz, 1H, H12), 7.06 – 7.04 (m, 2H, H11), 6.17 (d, J = 10.5 Hz, 1H, H6), 1.97 (s, 6H, CH_3). ^{13}C NMR (75.4 MHz, acetone-d6) δ: 182.2, 174.8, 138.7, 137.8, 136.4, 131.2, 131.1, 128.6, 128.2, 127.9, 127.0, 125.2, 20.7.

^1H NMR (400 MHz, acetone-d6)

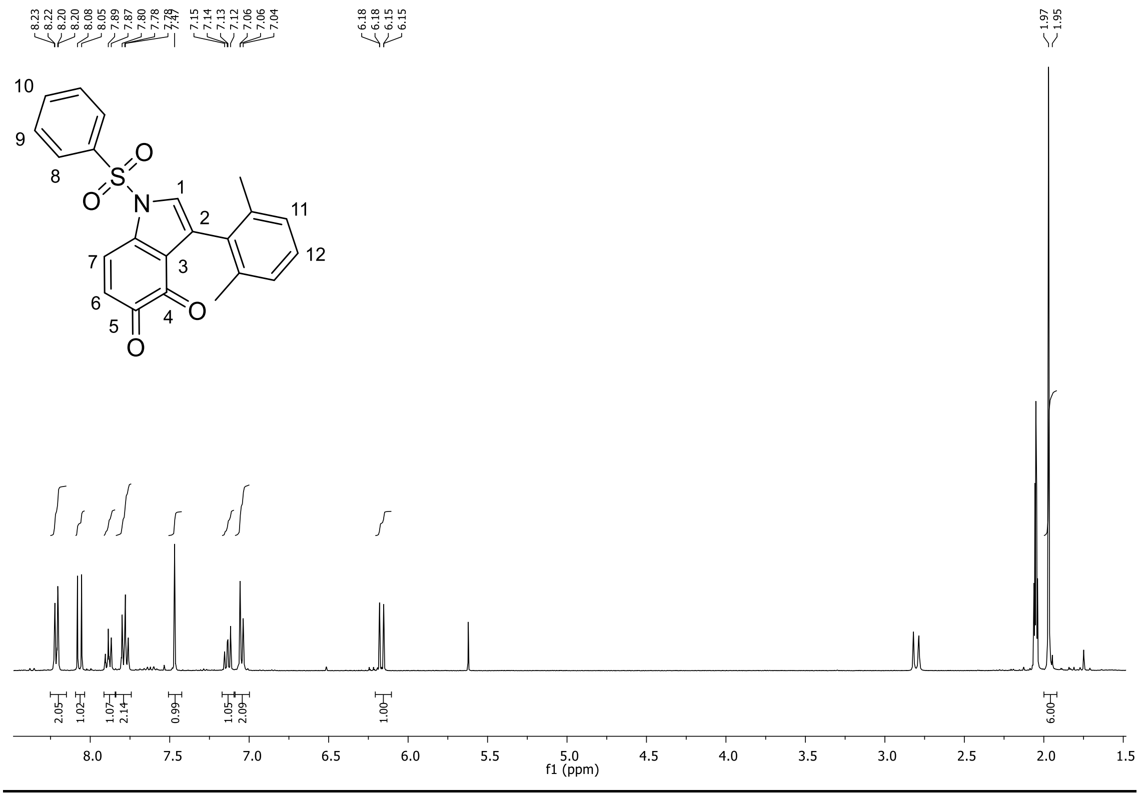

^{13}C NMR (75.4 MHz, acetone-d6)

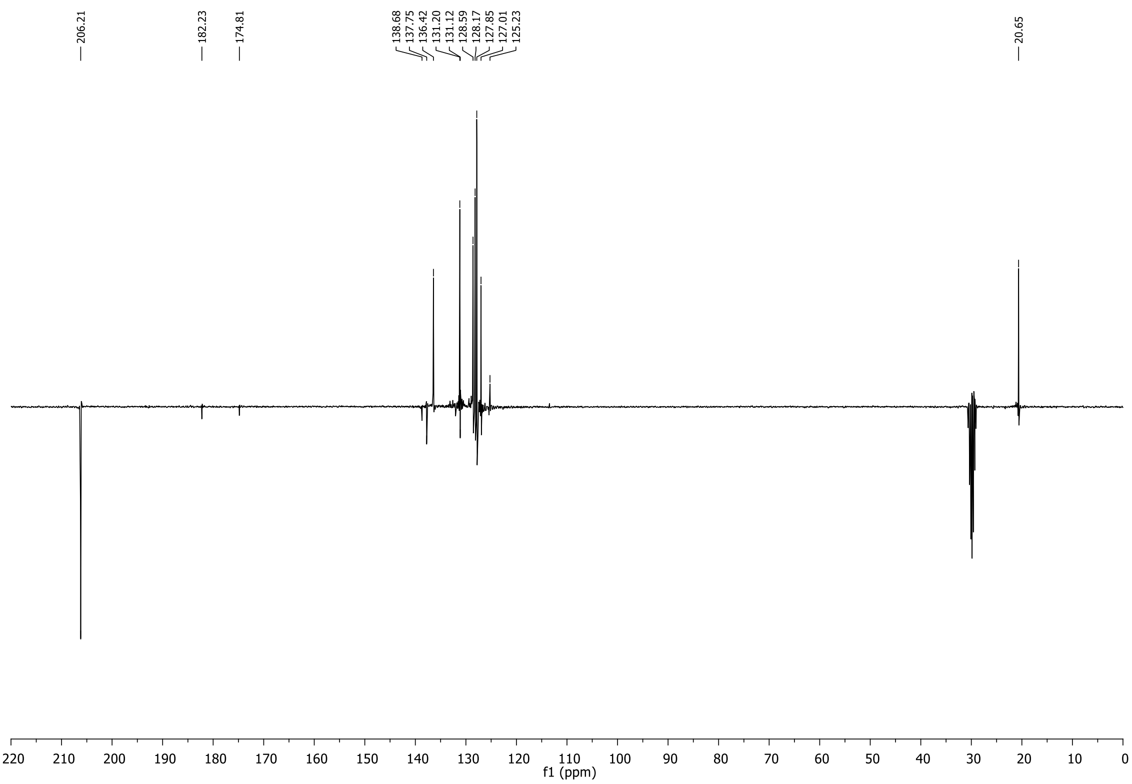

3-(2,6-dimethylphenyl)-7-((4-methoxyphenyl)thio)-1-(phenylsulfonyl)-1H-indole-4,5-diol **4red** / **TM7nred**.

Under argon atmosphere, a solution of 4-methoxybenzene-1-thiol (3.3 g, 23.6 mmol, 1.03 eq) in DMF (15 mL) was added under stirring to a solution of compound **9** (8.98 g, 22.9 mmol, 1 eq) in DMF, (100 mL) and the resulting mixture was stirred at rt for 1 hour. Then, the reaction mixture was poured into EtOAc and washed with water. The organic layer was dried over Na_2SO_4 and concentrated under reduced pressure. The residue was purified by column chromatography (eluent: 7:3 *n*-hexane/EtOAc) to obtain 8.5 g of target compound ***4red*** (16.0 mmol, 70% yield).

Due to its low stability and tendency to spontaneous oxidation to **4ox**, a full characterization of pure, diphenolic **4red** could not be carried out.

3-(2,6-dimethylphenyl)-7-((4-methoxyphenyl)thio)-1-(phenylsulfonyl)-1H-indole-4,5-dione **4ox** / **TM7nox**

IBX (95 mg, 0.34 mmol, 0.6 eq) was added to a stirred solution of **4red** (300 mg, 0.56 mmol, 1 eq) in DMF (2.4 mL) at rt. The reaction mixture was stirred and monitored by TLC (eluent: 6:4 *n*-hexane/EtOAc). After 30 minutes, distilled water (10 mL) was added and the aqueous phase was

Disease Models & Mechanisms • Supplementary information

extracted with EtOAc (3 x 50 mL); the collected organic phases were washed with a saturated solution of $NaHCO_3$ (1 x 70 mL) and distilled water (1 x 70 mL).After drying over Na_2SO_4 and filtering, the solvent was evaporated under reduced pressure. The crude, dark blue solid was purified by flash chromatography (eluent: 7:3 *n*-hexane/EtOAc), affording 238 mg of **4ox** as a dark blue solid (80% yield). MS (ESI^+): m/z 530.27 [M + H^+]. Calculated MS for $C_{29}H_{23}NO_5S_2$: 529.10 (ox); m/z 532.36 [M + H^+]. Calculated MS for $C_{29}H_{25}NO_5S_2$: 531.12 (red). By LC-MS analysis, compound **4** can be detected both in its oxidized (**4ox**) and reduced (**4red**) form, due to the presence of TFA in the HPLC solvents which promotes the formation of the less stable reduced form. ^1H NMR (400 MHz, acetone-d6) δ 7.89 – 7.83 (m, 1H, H10), 7.75 (m, 4H, H8, H9), 7.59 – 7.55 (m, 2H, H13), 7.33 – 7.29 (m, 2H, H14), 7.26 (s, 1H, H1), 7.12 (dd, J = 6.8 Hz, J = 8.2 Hz, 1H, H12), 7.05 – 70.3 (m, 2H, H11), 6.85 (s, 1H, H6), 3.98 (s, 3H, OCH_3), 1.99 (s, 6H, CH_3). ^{13}C NMR (100.6 MHz, acetone-d6) δ 177.2, 173.7, 161.8, 141.3, 137.6, 136.9, 135.4, 131.2, 130.2, 127.7, 127.1, 126.9, 126.1, 125.0, 122.6, 120.0, 118.9, 116.0, 55.2, 19.7.

^1H NMR (400 MHz, acetone-d6)

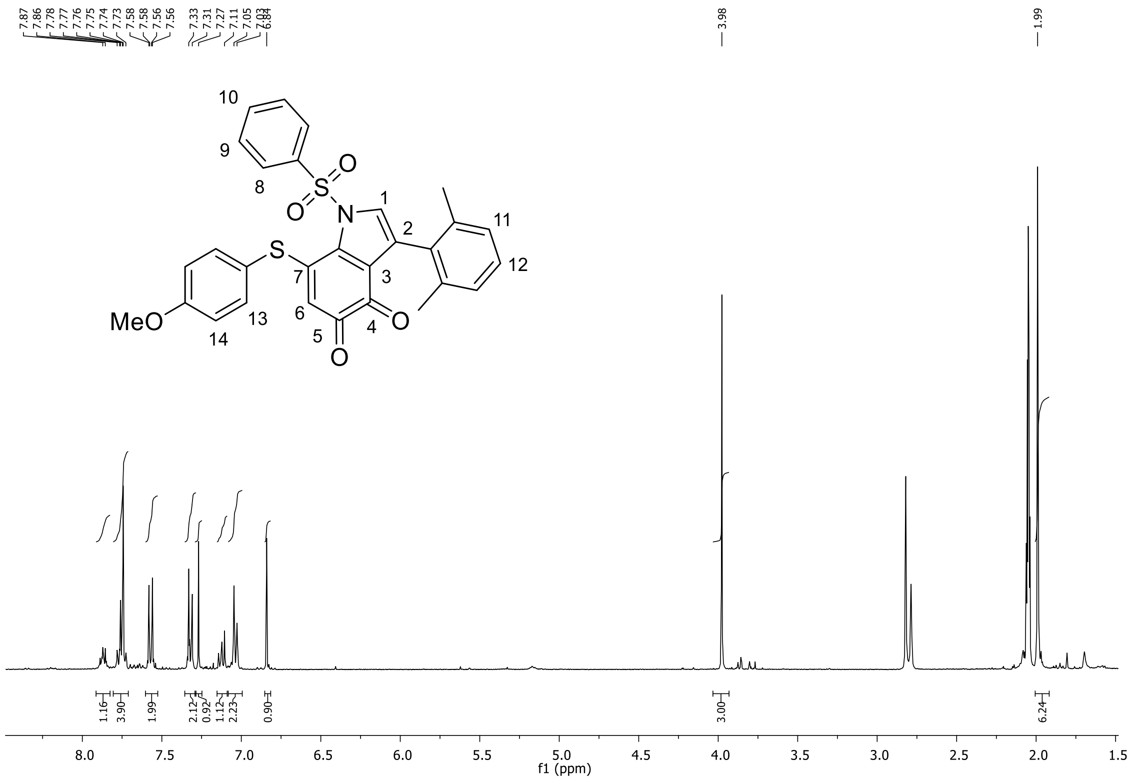

^{13}C NMR (100.6 MHz, acetone-d6)

Disease Models & Mechanisms • Supplementary information

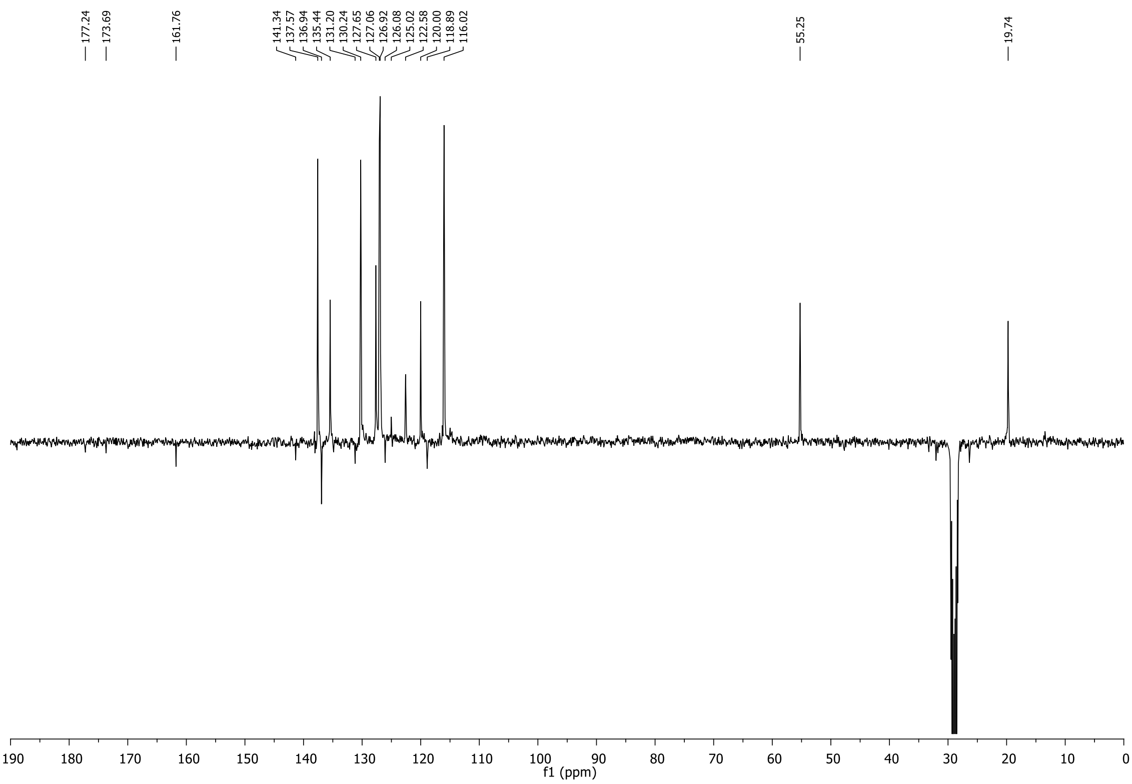

3-(2,6-dimethylphenyl)-7-((4-methoxyphenyl)thio)-1-(phenylsulfonyl)-1H-indole-4,5-diyl diacetate **5 / TM8n**.

Previously disareated acetic anhydride (0.072 mL, 0.76 mmol, 4 eq) was added under nitrogen atmosphere at rt to a stirred solution of compound **4red** (100 mg, 0.19 mmol, 1 eq) in dry DCM (1.9 mL), then after cooling to 0°C pyridine (0.153 mL, 1.9 mmol, 10 eq) was added dropwise. The reaction was stirred at rt monitoring by TLC (eluent: 95:5 DCM/MeOH) for ca 24 h. After reaction completion, distilled water (10 mL) was added to the reaction mixture. The mixture was extracted with DCM (3 x 20 mL) and the organic phase was washed with 1 M HCl (1 x 60 mL), brine (1 x 60 mL) and dried over Na_2SO_4. Finally, the solvent was evaporated under reduced pressure affording 113 mg of pure **5** as a white solid (0.18 mmol, 97% yield). MS (ESI^+): *m/z* 616.56 [M + H^+]. Calculated MS for $C_{33}H_{29}NO_7S_2$: 615.14. ^1H NMR (400 MHz, acetone-d6) δ(ppm): 7.78 – 7.76 (m, 2H, H8), 7.73 – 7.69 (m, 1H, H10), 7.63 – 7.60 (m, 2H, H9), 7.58 – 7.54 (m, 3H, H1, H9), 7.45 (s, 1H, H6), 7.23 – 7.18 (m, 3H, H12, H13), 7.12 (d, *J* = 7.7 Hz, 2H, H11), 3.95 (s, 3H, OCH_3), 2.25 (s, 3H, $OCOCH_3$), 1.92 (s, 6H, CH_3), 1.33 (s, 3H, $OCOCH_3$). ^{13}C NMR (101 MHz, Acetone) δ 168.3, 168.1, 162.0, 139.1, 138.0, 137.6, 137.4, 135.5, 134.5, 132.56, 132.1, 130.6, 128.9, 127.9, 127.8, 126.1, 123.13, 122.8, 120.9, 116.5, 111.7, 56.0, 20.7, 20.1, 18.7.

^1H NMR (400 MHz, acetone-d6)

Disease Models & Mechanisms • Supplementary information

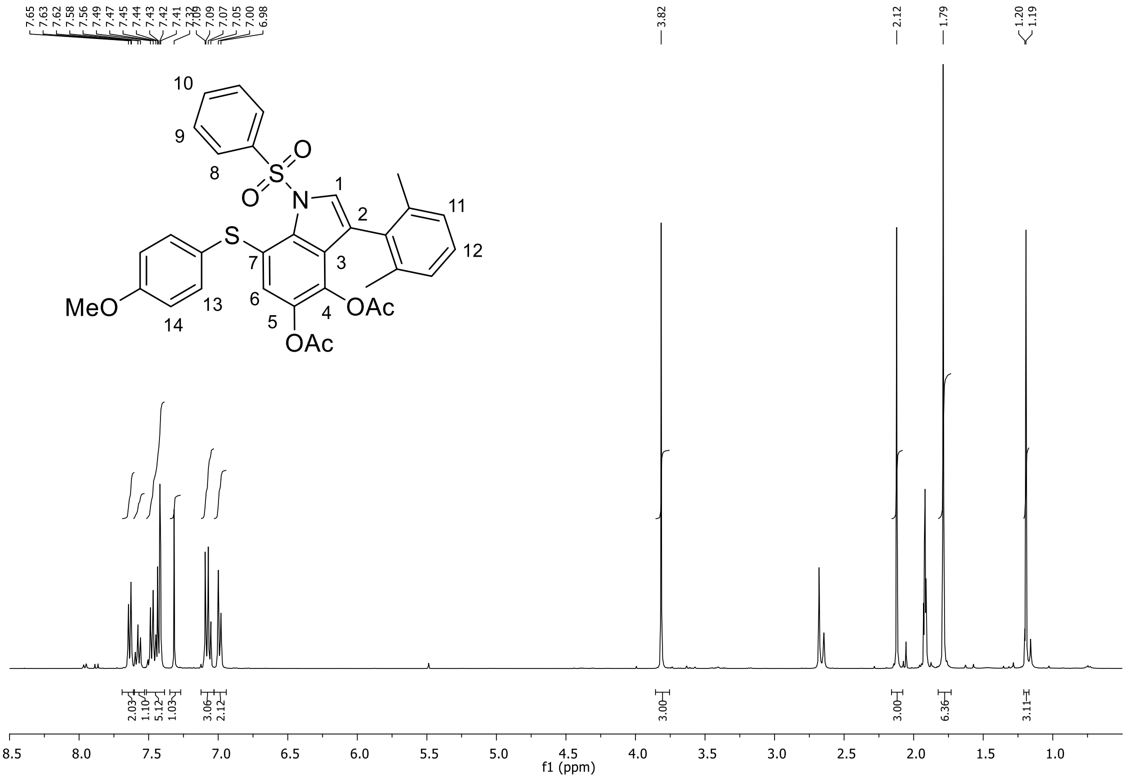

^{13}C NMR (100.6 MHz, Acetone)

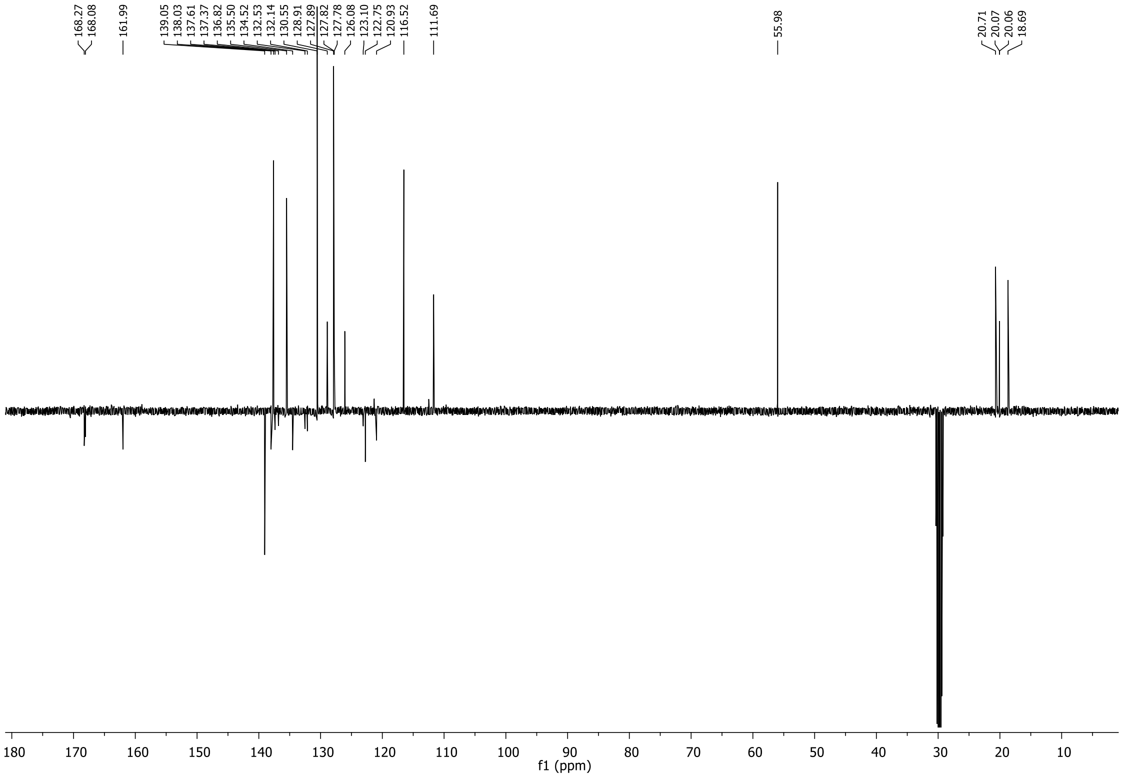

Analytical Solubility Method

Kinetic solubility is determined by preparing a concentrated stock solution of 10 mM of the analyte in organic polar solvent DMSO of the compound that is diluted to 200 µM in an aqueous

Disease Models & Mechanisms • Supplementary information

buffer (eg. PBS). The solubility is estimated by LC-MS analysis of a solution obtained after filtration or spin-down to remove the insoluble part. The column used was a reverse phase 18, ACQUITY UPLC BEH SHIELD, RP18, with Phase A: Water (Merck) with 0,05% Trifluoroacetic Acid (Sigma), Phase B: Acetonitrile (Merck) with 0,05% Trifluoroacetic Acid (Sigma). Data were analyzed with Masslynx 4.0. The Kinetic Solubility (µM) was calculated according to =

$$200\ \mu M \times \frac{(\text{Peak area})_{\text{Buffer}}}{(\text{Peak area})_{\text{DMSO}}} \times \frac{(\text{Injection volume})_{\text{DMSO}}}{(\text{Injection volume})_{\text{Buffer}}}$$

Bibliography

(1) Manzoni, L.; Zucal, C.; Maio, D. Di; D'Agostino, V. G.; Thongon, N.; Bonomo, I.; Lal, P.; Miceli, M.; Baj, V.; Brambilla, M.; Cerofolini, L.; Elezgarai, S.; Biasini, E.; Luchinat, C.; Novellino, E.; Fragai, M.; Marinelli, L.; Provenzani, A.; Seneci, P. Interfering with HuR-RNA Interaction: Design, Synthesis and Biological Characterization of Tanshinone Mimics as Novel, Effective HuR Inhibitors. *J. Med. Chem.* **2018**, *61* (4), 1483–1498. https://doi.org/10.1021/acs.jmedchem.7b01176.

Disease Models & Mechanisms • Supplementary information
